# Digital Health Literacy: Bibliometric Analysis

**DOI:** 10.2196/35816

**Published:** 2022-07-06

**Authors:** Keng Yang, Yekang Hu, Hanying Qi

**Affiliations:** 1 Institute of Economics Tsinghua University Beijing China; 2 One Belt-One Road Strategy Institute Tsinghua University Beijing China; 3 China National Health Development Research Center Beijing China; 4 The New Type Key Think Tank of Zhejiang Province “Research Institute of Regulation and Public Policy” Zhejiang University of Finance and Economics Hangzhou China; 5 China Institute of Regulation Research Zhejiang University of Finance and Economics Hangzhou China

**Keywords:** digital health literacy, eHealth, digital divide, bibliometrics, VOSviewer, CiteSpace

## Abstract

**Background:**

Digital health is growing at a rapid pace, and digital health literacy has attracted increasing attention from the academic community.

**Objective:**

The purposes of this study are to conduct a systematic bibliometric analysis on the field of digital health literacy and to understand the research context and trends in this field.

**Methods:**

Methods: A total of 1955 scientific publications were collected from the Web of Science core collection. Institutional co-operation, journal co-citation, theme bursting, keyword co-occurrence, author co-operation, author co-citation, literature co-citation, and references in the field of digital health literacy were analyzed using the VOSviewer and CiteSpace knowledge mapping tools.

**Results:**

The results demonstrate that the United States has the highest number of publications and citations in this field. The University of California System was first in terms of institutional contributions. The *Journal of Medical Internet Research* led in the number of publications, citations, and co-citations. Research areas of highly cited articles in the field of digital health literacy mainly include the definition and scale of health literacy, health literacy and health outcomes, health literacy and the digital divide, and the influencing factors of health literacy.

**Conclusions:**

We summarized research progress in the field of digital health literacy and reveal the context, trends, and trending topics of digital health literacy research through statistical analysis and network visualization. We found that digital health literacy has a significant potential to improve health outcomes, bridge the digital divide, and reduce health inequalities. Our work can serve as a fundamental reference and directional guide for future research in this field.

## Introduction

With the development of digital technology, big data, the Internet of Things, artificial intelligence, cloud computing, wearable devices, and so on, digital technologies are constantly being applied to the medical and health fields, giving new vitality to the development of medical health. The World Health Organization (WHO) defines digital health as “the field of knowledge and practice associated with any aspect of adopting digital technology to improve health, from inception to operation”[1]. Digital health expands the concept of eHealth by including other uses of digital technology in health areas such as the Internet of Things, advanced computing, big data analysis, and artificial intelligence [1]. The US Food and Drug Administration (FDA) defines digital health as having “a broad scope which includes mobile health (mHealth), health information technology, wearable devices, telehealth and telemedicine, and personalized medicine” [2].

As digital health has become more common, digital literacy and health literacy have become important determinants of the usefulness of digital health technologies [3]. Digital literacy refers to “the ability to use information and communication technologies to find, evaluate, create, and communicate information, requiring both cognitive and technical skills” [4]. Health literacy is defined by WHO as “the cognitive and social skills which determine the motivation and ability of individuals to gain access to, understand, and use information in ways which promote and maintain good health” [5]. Based on this, the concept of digital health literacy is gradually taking shape and is receiving more research attention. It has been argued that digital health literacy is a superdeterminant of health that depends on 3 key factors: civic, digital, and health literacy [4]. According to the existing literature, the definition of digital health literacy refers to the application of the definition of health literacy to digital contexts and environments [6]. Therefore, we define digital health literacy in this study as the ability to seek, find, understand, and appraise health information from electronic sources or digital contexts and apply the knowledge gained to addressing or solving a health problem [6,7].

In this study, the concept of digital health literacy also includes eHealth literacy. We argue that the concept of digital health literacy grew from eHealth literacy and was honed thereafter. Norman [8] defined eHealth literacy in 2006 as the ability to seek, discover, understand, and evaluate health information through electronic channels and to apply the knowledge gained to solve health problems. From a definitional perspective, the concepts of digital health literacy and eHealth literacy are similar. Only the channel of accessing and processing health information has changed, evolving from the earlier electronic channel to the digital technology channel. In the existing literature, digital health literacy is often used interchangeably with eHealth literacy [6,9-11]. Specifically, the relationship between digital health literacy and eHealth literacy represents an evolving process of the same thing. With the innovative development of digital technology, eHealth technology is gradually evolving into digital health technology. According to the WHO report, digital health expands and encompasses eHealth [1], and eHealth literacy has become more popular in the era of eHealth technology. With the development of digital health technologies, the term digital health literacy has received increasingly general attention. Taking digital health literacy measurement as an example, early scholars, with Norman [8] at the core, heavily investigated how to measure eHealth literacy. Norman believed that eHealth literacy contains 6 core literacies: traditional literacy, health literacy, information literacy, science literacy, media literacy, and computer literacy. In 2006, Norman developed the eHealth Literacy Scale (eHEALS), which has since been widely used in research on eHealth literacy [12]. The eHEALS is gradually being extended through multinational versions in Italian [13], Spanish [14], German [15], Korean [16], and so on. As research deepens, digital health literacy assessment tools are continually iterated and improved by scholars to enhance the measurement of digital health literacy [6,17,18].

Health literacy is an independent and intermediary determinant of health [19], and improving health literacy helps to improve health outcomes [20,21]. Accordingly, digital health literacy is also a determinant of health [4]. Improving digital health literacy at the population level could address health inequalities, the digital divide [22,23], and public attitudes toward as well as practices and awareness of digital health [1]. That is, improving digital health literacy is a useful solution to emerging health challenges. This has become especially clear during the COVID-19 pandemic, with digital health literacy being a key capability for finding information on COVID-19 on the internet [24]. Therefore, it is important to understand the current situation and trends in digital health literacy research and to identify future research opportunities.

Moreover, there is some bibliometric research related to health literacy and eHealth literacy [25-27]. However, although these studies have done systematic bibliometrics, their core concepts are limited to eHealth literacy. They were unable to comprehensively embrace cutting-edge developments in the field of digital health literacy. In addition to including eHealth literacy, we further encompassed literature on the intersection of digital technology and health literacy and that of digital health and health literacy to expand and enrich the existing research.

Against this background, we use bibliometric analysis and knowledge network visualization to analyze institutional co-operation, journal co-citation, topic bursting, keyword co-occurrence, author co-operation, author co-citation, reference co-citation, and bursting in the field of digital health literacy. In this way, we map the knowledge structure of research in this field, along with research trends and trending topics. To the best of our knowledge, this is the first comprehensive bibliometric analysis in digital health literacy, and this study can provide basic support and directional guidance for future research in this field.

## Methods

### Data Collection

**Figure 1:**
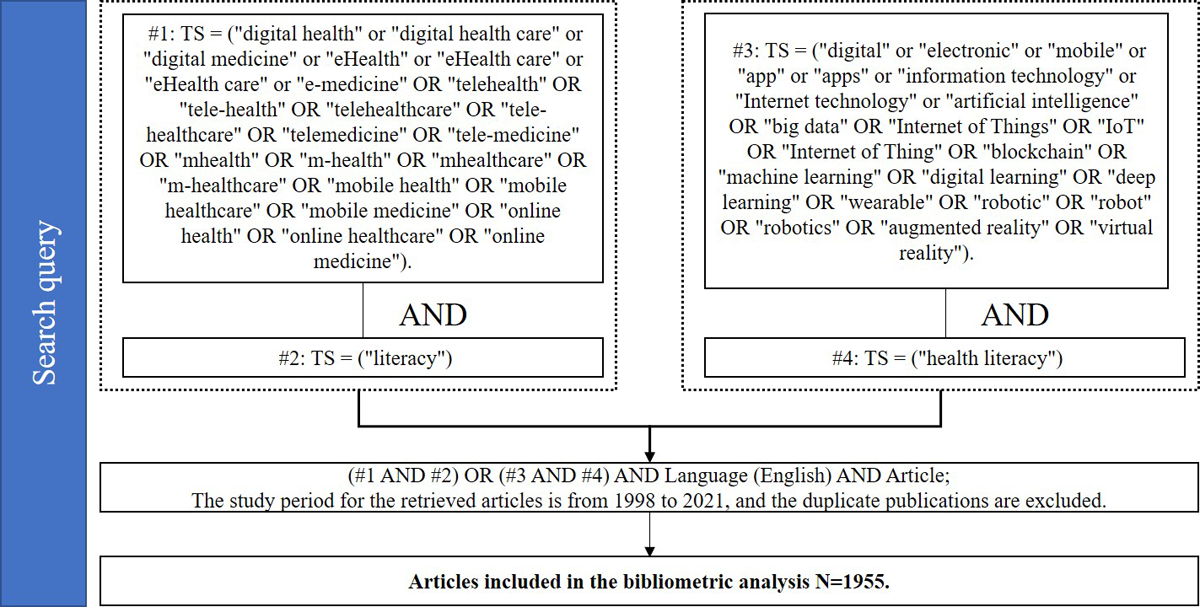
The flowchart for data collection.

The Web of Science (WoS) Core Collection served as the data source in this study. This database includes journals that are indexed in the Social Science Citation Index (SSCI), the Science Citation Index Extension (SCIE), and the Art and Humanities Citation Index (A&HCI). The retrieval method relied on the steps outlined by Chen [28]. We retrieved articles containing both the keywords “digital health” and “literacy” as well as those with the keyword “health literacy” and keywords related to digital technology. Our specific screening strategies are shown in Figure 1 and outlined as follows.

First, we retrieved articles with the keyword “digital health” and derivative keywords of the topic. The query command for #1 was TS = (“digital health” OR “digital health care” OR “digital medicine” or “eHealth” OR “eHealth care” OR “eHealth care” OR “e-medicine” OR “telehealth” OR “tele-health” OR “telehealthcare” OR “tele-healthcare” OR “telemedicine” OR “tele-medicine” OR “mHealth” OR “m-health” OR “mHealthcare” OR “m-healthcare” OR “mobile health” OR “mobile healthcare” OR “mobile medicine” OR “online health” OR “online healthcare” OR “online medicine”). TS refers to the topic tag for search string retrievals.

Second, we retrieved articles with the keyword “literacy.” The query command for #2 was TS = (“literacy”).

Third, we retrieved articles with digital technology–related keywords. The query command for #3 was TS = (“digital” OR “electronic” OR “mobile” OR “app” OR “apps” OR “information technology” OR “Internet technology” OR “artificial intelligence” OR “big data” OR “Internet of Things” OR “IoT” OR “Internet of Thing” OR “blockchain” OR “machine learning” OR “digital learning” OR “deep learning” OR “wearable” OR “robotic” OR “robot” OR “robotics” OR “augmented reality” OR “virtual reality”).

Fourth, we retrieved articles with the keyword “health literacy.” The query command for #4 was TS = (“health literacy”).

Fifth, we combined the commands above[(#1 AND #2) OR (#3 AND #4)] AND Language (English) AND Article. This was the last step of the screening process. The query command (#1 AND #2) indicates retrieving papers related to both digital health and literacy. This refers to articles that study literacy in the field of digital health. The second query command for the last step (#3 AND #4) indicates retrieving articles related to both digital technology and health literacy. This refers to articles that focused on digital technology in the field of health literacy. Therefore, the combined command of the last step aimed to retrieve articles in English related to “digital health and literacy” or “digital technology and health literacy.”

A total of 1955 articles in English were retrieved as research samples. The beginning time point for data collection was 1990, and the study period for the retrieved articles was from 1998 to 2021. The data retrieval took place on September 30, 2021.

### Analysis Methodologies

bibliometric analysis, or bibliometrics, was first described by Pritchard [29] as “the application of mathematical and statistical methods to articles and other forms of communication.” Bibliometrics is a method of information analysis that measures research trends and knowledge structures in a field to obtain quantifiable, objective data [30]. It has been extensively used to quantitative analyze academic literature to describe trending topics and contributions of scholars, journals, and countries [31] and help researchers understand the current research trends, distribution, and core topics in a given field [32].

We used scientific mapping tools for bibliometric analysis. Currently popular tools include VOSviewer [33], CiteSpace [34], BibExcel [35], HistCite [35], and others. We selected VOSviewer and CiteSpace as our analysis tools. The reason for this was that VOSviewer has better visualization in network and cluster analysis, and CiteSpace is better in literature timeline analysis. A combination of these two tools can better achieve our research goals. VOSviewer was developed by Van Eck and Waltman [33] and features a powerful bibliometric maps function that can clearly visualize the network of literature, keywords, authors, and so on. Using VOSviewer (version 1.6.16), we drew diagrams for institutional co-operation, journal co-citation, keyword co-occurrence, author co-operation, author co-citation, and literature co-citation. CiteSpace was developed by Chen [34] to implement 2 complementary visualizations: cluster view and time zone view. We used CiteSpace (5.8.R1) to draw 2 analysis mappings: theme bursting and reference bursting. For the distribution of publications, countries, institutions, journals, and authors, Microsoft Excel was employed to perform the analyses.

## Results

### Overall Trends in Publications and Citations

**Figure 2:**
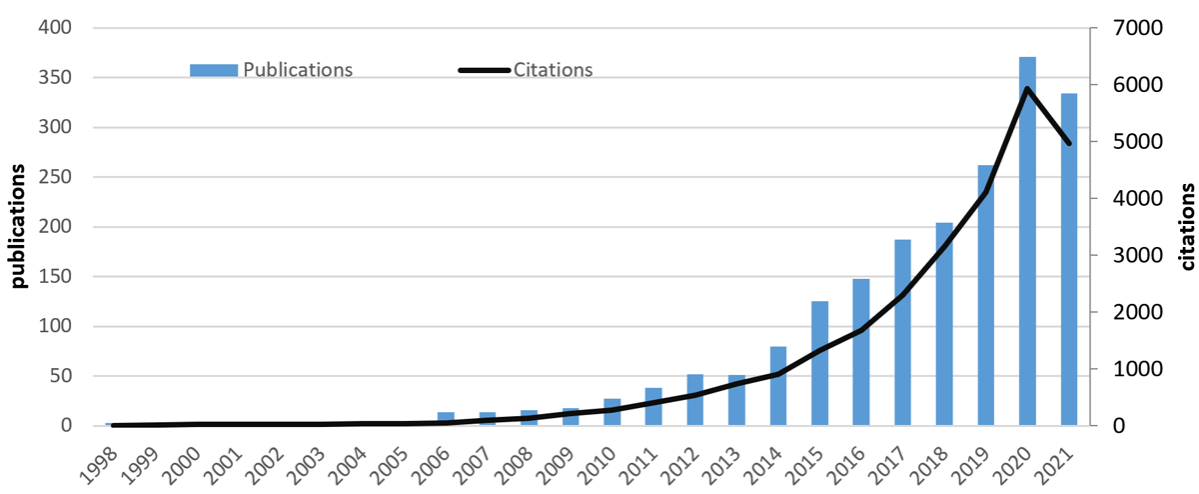
Total publications and citations from 1998 to 2021.

Figure 2 depicts trends in publications and citations in digital health literacy. Our search yielded 1955 articles covering the period from 1998 to 2021. From when the first article was published in 1998 until 2021, the number of articles published continuously increased, with an obvious overall growth trend. In terms of average annual publications, the trend can be divided into 3 stages: (1) 1998 to 2005 was the gestation period, and the number of publications stayed in the single digits, with an average of 3 articles per year; (2) 2006 to 2013 was a slow growth period, and the number of publications remained below 100 each year. The average number of publications was about 34 annually; (3) Since 2014, digital health literacy research has undergone rapid growth. As of September 30, 2021, the average annual number of articles was 233, with a peak in 2020 (n=392, 20.05%). Publications increased dramatically from 2014 to 2021, accounting for 87.5% (1711/1955) of all publications, and are still increasing. Citations have a similar overall development trend.

### Country Distribution

About 91.7% (1792/1955) of the publications were from the top 10 productive countries or regions shown in Table 1. The United States ranked first with 913 published articles, accounting for 46.7% of all 1955 publications and far exceeding the numbers of other countries. Australia was second (198/1955, 10.1%), followed by the United Kingdom (136/1955, 7%), Canada (108/1955, 5.5%), China (104/1955, 5.3%), and Germany (102/1955, 5.2%).

**Table 1:** Top 10 publication countries/regions (N=1955).

Countries/regions	Publications, n (%)
United States	913 (47)
Australia	198 (10.1)
England	136 (7)
Canada	108 (5.5)
China	104 (5.3)
Germany	102 (5.2)
Netherlands	99 (5.1)
Switzerland	45 (2.3)
Denmark	44 (2.3)
Sweden	43 (2.2)

### Institution Distribution

The top 10 research institutions according to publication number are presented in Table 2. The institution that published the most papers at 100 publications was the University of California System, accounting for 5.12% (n=1955) of all publications. Harvard University was second, with 64 (3.3 %) publications, and the State University System of Florida came in third, with 3.2% (n=1955) of all publications.

Of the top 10 institutions, 1 is a government agency: the US Department of Veterans Affairs, which is the administrative department for veterans and mainly focuses on health literacy among veterans. The other 9 institutions are all universities.

**Figure 3:**
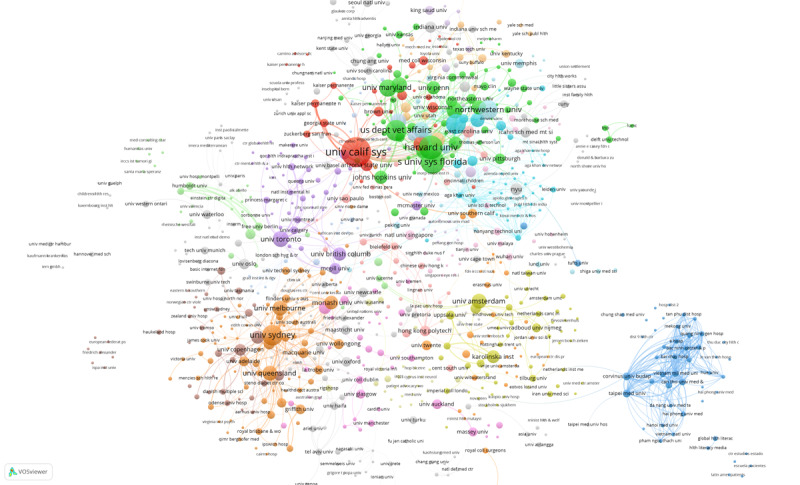
Institutional coauthorship network (1998-2021).

**Figure 4:**
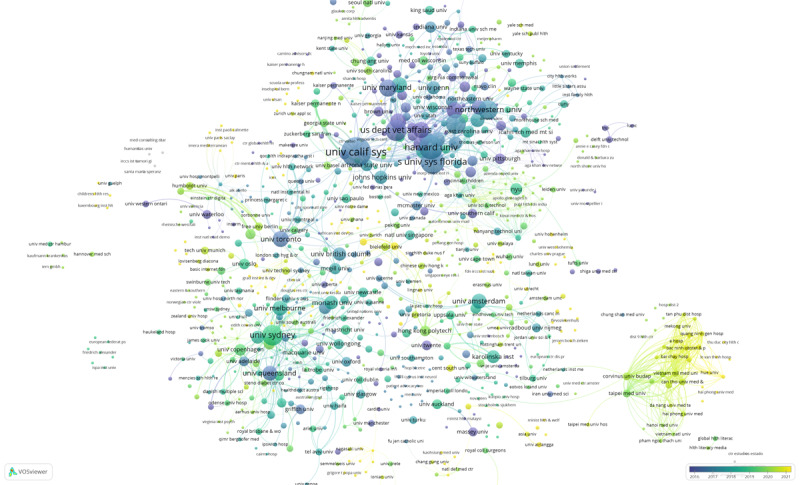
Time trend map of organizations' coauthorship.

We also analyzed coauthorship among institutions. A total of 959 institutions with high association strength were automatically identified using VOSviewer and were used to draw the institutional co-operation network shown in Figures 3 and 4. A total of 31 clusters were formed, and different colors represent different clusters. In these figures, node size refers to publications, circle color to clustering, and link thickness to co-operation strength. The minimum number of documents of an institution is 1 publication. The minimum number of citations of an institution is 0. The largest cluster has 80 institutions, and the smallest has 7. We clustered the institutions network using the default VOS clustering, which uses a clustering algorithm similar to modularity-based clustering. Of these clusters, 4 stand out in comparison to others, and these co-operation networks have obvious geographical characteristics. In Figure 3 (see Multimedia Appendix 1 for full-size image), the red cluster is a collaborative network with the University of California System at its core, while the green cluster is a collaborative network with Harvard University, the State University System of Florida, and the US Department of Veterans Affairs as its core. Institutions in these 2 clusters are primarily from the United States. The thick line between the University of California System and Harvard University suggests a high level of collaboration. The orange cluster is a collaborative network with the University of Sydney, University of Melbourne, and University of Queensland as the core. These institutions are mainly from Australia and the Commonwealth. The main institutions in the blue cluster (lower right corner) are largely based in emerging market countries such as Hungary, Turkey, and Vietnam, and they mainly co-operate with other institutions also located in emerging markets.

Figure 4 shows the average publication year of articles published by each institution (See Multimedia Appendix 2 for full-size image). Time is represented by different colors. The darker the color, the earlier the average publication year of the institution. As seen, the average publishing year of institutions from the United States was earlier, followed by institutions from Australia. The average publishing year of institutions from emerging markets was closer to 2021; this indicates that research attention to digital health literacy has gradually spread from high-income countries to emerging market countries and regions.

**Table 2:** Top 10 institutions of publications.

Institution	Publications, n (%)
University of California System	100 (5.1)
Harvard University	64 (3.3)
State University System of Florida	63 (3.2)
University of North Carolina	49 (2.5)
University of Sydney	49 (2.5)
US Department of Veterans Affairs	49 (2.5)
University of Texas System	44 (2.3)
Northwestern University	42 (2.2)
University System of Maryland	37 (1.9)
University of London	33 (1.7)

### Journal Distribution

The 1955 citing articles in this paper were published in a total of 710 different journals. Table 3 shows the top 10 journals in terms of publication number and reports both their total citation frequency and average citation frequency. The *Journal of Medical Internet Research* ranked first with 207 publications, accounting for 10.6% of all 1955 articles, and it had the highest citation frequency at 6663 citations, accounting for 24.7% of all articles’ total citations (N=27,012). Two other journals from the same publisher (JMIR Publications) also ranked highly in terms of publications, namely *JMIR mHealth and uHealth* (62/1955, 3.2%) and *JMIR Research Protocols* (39/1955, 2%), indicating that JMIR Publications paid more attention to digital health literacy research within the study period.

Table 3 shows that when journals are ranked according to articles published, their citation numbers are not consistent with their ranking. Some journals have relatively high citation numbers even though they have published relatively few articles. Average citation numbers illustrate that certain journals have higher average citation numbers than others, which may reflect the relative quality of these journals’ publications.

Table 4 presents the top 10 journals ranked by citation number. As the table indicates, some journals have higher citation numbers despite having fewer publications. For example, the Journal of General Internal Medicine has the second highest number of citations (n=894, 3.3%) but only published 9 papers with an average of 99.33 citations, indicating that the papers published in this journal have played a significant role in promoting digital health literacy research. Annals of Internal Medicine had the highest average citation numbers (n=155, 0.58%) with only 3 publications; the total number of citations for this journal was 465(1.7%), indicating that the 3 publications were relatively important and made significant contributions to digital health literacy research.

**Table 3:** Top 10 most productive journals.

Journals	Publications^a^, n (%)	Citations^b^, n (%)	Average citations, n
*Journal of Medical Internet Research*	207 (10.6)	6663 (24.7)	32.19
*JMIR mHealth and uHealth*	62 (3.2)	567 (2.1)	9.15
*International Journal of Environmental Research and Public Health*	46 (2.4)	218 (0.8)	4.74
*JMIR Research Protocols*	39 (2)	187 (0.7)	4.79
*Patient Education and Counseling*	37 (1.9)	679 (2.5)	18.35
*BMC Public Health*	31 (1.6)	536 (2)	17.29
*Telemedicine and e-Health*	29 (1.5)	404 (1.5)	13.93
*BMC Medical Informatics and Decision Making*	26 (1.3)	372 (1.4)	14.31
*BMJ Open*	26 (1.3)	130 (0.5)	5
*Journal of Health Communication*	24 (1.2)	612 (2.3)	25.50

^a^N=1955.

^b^N=27,012.

**Table 4:** Top 10 journals according to number of citations.

Journal	Citations^a^, n (%)	Publications^b^, n (%)	Average references, n
*Journal of Medical Internet Research*	6663 (24.7)	207 (10.6)	32.19
*Journal of General Internal Medicine*	894 (3.3)	9 (0.5)	99.33
*Journal of the American Medical Informatics Association*	702 (2.6)	19 (1.0)	36.95
*Patient Education and Counseling*	679 (2.5)	37 (1.9)	18.35
*Journal of Health Communication*	612 (2.3)	24 (1.2)	25.50
*JMIR mHealth and uHealth*	567 (2.1)	62 (3.7)	9.15
*BMC Public Health*	536 (2.0)	31 (1.6)	17.29
*BMC Health Services Research*	532 (2.0)	18 (0.9)	29.56
*Annals of Internal Medicine*	465 (1.7)	3 (0.2)	155
*Telemedicine and e-Health*	404 (1.5)	29 (1.5)	13.93

^a^N=27,012.

^b^N=1955.

**Figure 5:**
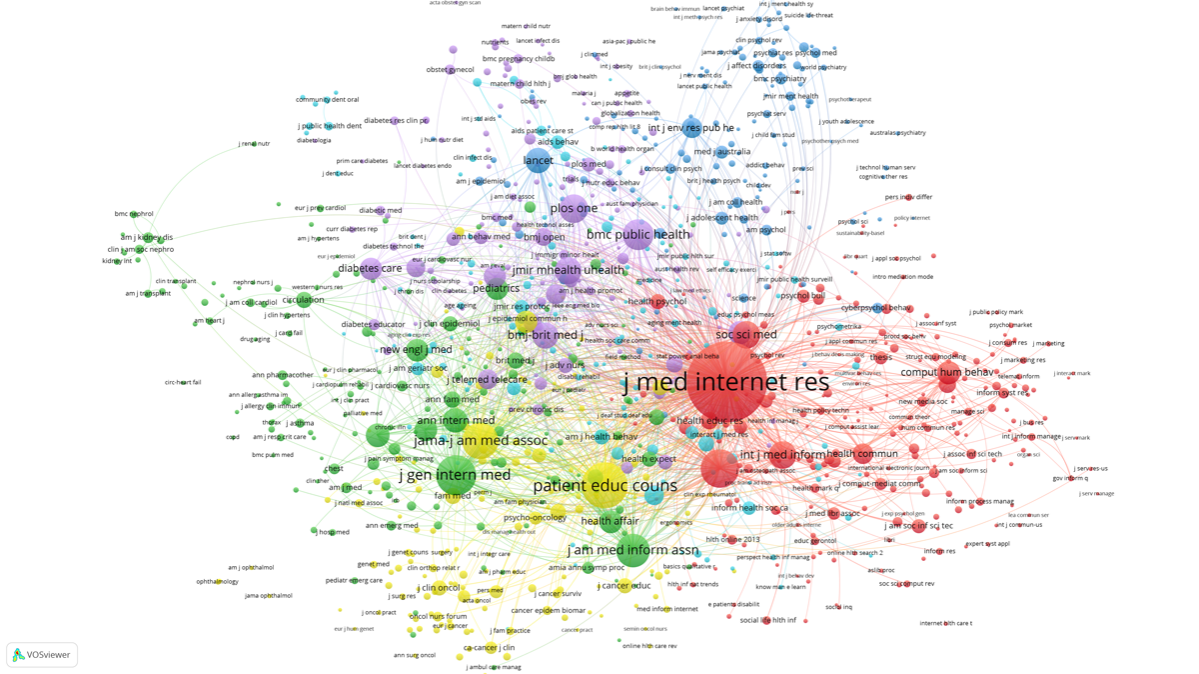
The co-citation network of journals.

We conducted a co-citation analysis of journals based on the references of 1955 articles. We used VOSviewer to form a journal co-citation network with 6 clusters, as presented in Figure 5. In this figure, color represents cluster, circle size represents the outgoing document quantity, and line thickness represents co-citation intensity (See Multimedia Appendix 3 for full-size image). The co-citation network consists of 1000 journals, and each journal has more than 10 citations and strong co-citation correlation. As indicated by the largest red circle in the figure, the *Journal of Medical Internet Research* is at the core of the whole co-citation network, and it has the largest number of citations and greatest co-citation intensity. It is a leading journal in digital health literacy research and focuses mainly on fields such as health informatics and digital health. In addition, the *Journal of Health Communication*, which features in the same red cluster, is also important, indicating that their co-citation relationships are relatively strong. This journal also focuses on health informatics.

In the yellow cluster, *JAMA: Journal of the American Medical Association* is important. It is a top journal in the field of medicine and is the most widely circulated general medical journal in the world. *Patient Education and Counseling*, which focuses mainly on patient education and health promotion, is also important in the yellow cluster.

The most important journals in the green cluster are *Journal of General Internal Medicine* and *Journal of the American Medical Informatics Association*. Meanwhile, *BMC Public Health* and *Public Library of Science (PLOS) One* stand out in the purple cluster, and *The Lancet* leads in the blue cluster. All these journals have played an important supporting role in digital health literacy research.

### Research Topics

We investigated the research topics of digital health literacy in terms of both research categories and research themes. Table 5 shows the top 10 categories of digital health literacy publications. The unit of measurement in the table is the number of publications. The data for these research categories are generated by the WoS database system based on search results. Health care sciences services (569/1955, 29.1%), medical informatics (436/1955, 22.3%), and public environmental occupational health (427/1955, 21.8%) were the 3 most important research categories for digital health literacy. In addition, digital health literacy also covers “health policy services,” “information science library science,” “nursing,” “medicine general internal,” “oncology,” “computer science information systems,” and “communication.”

**Figure 6:**
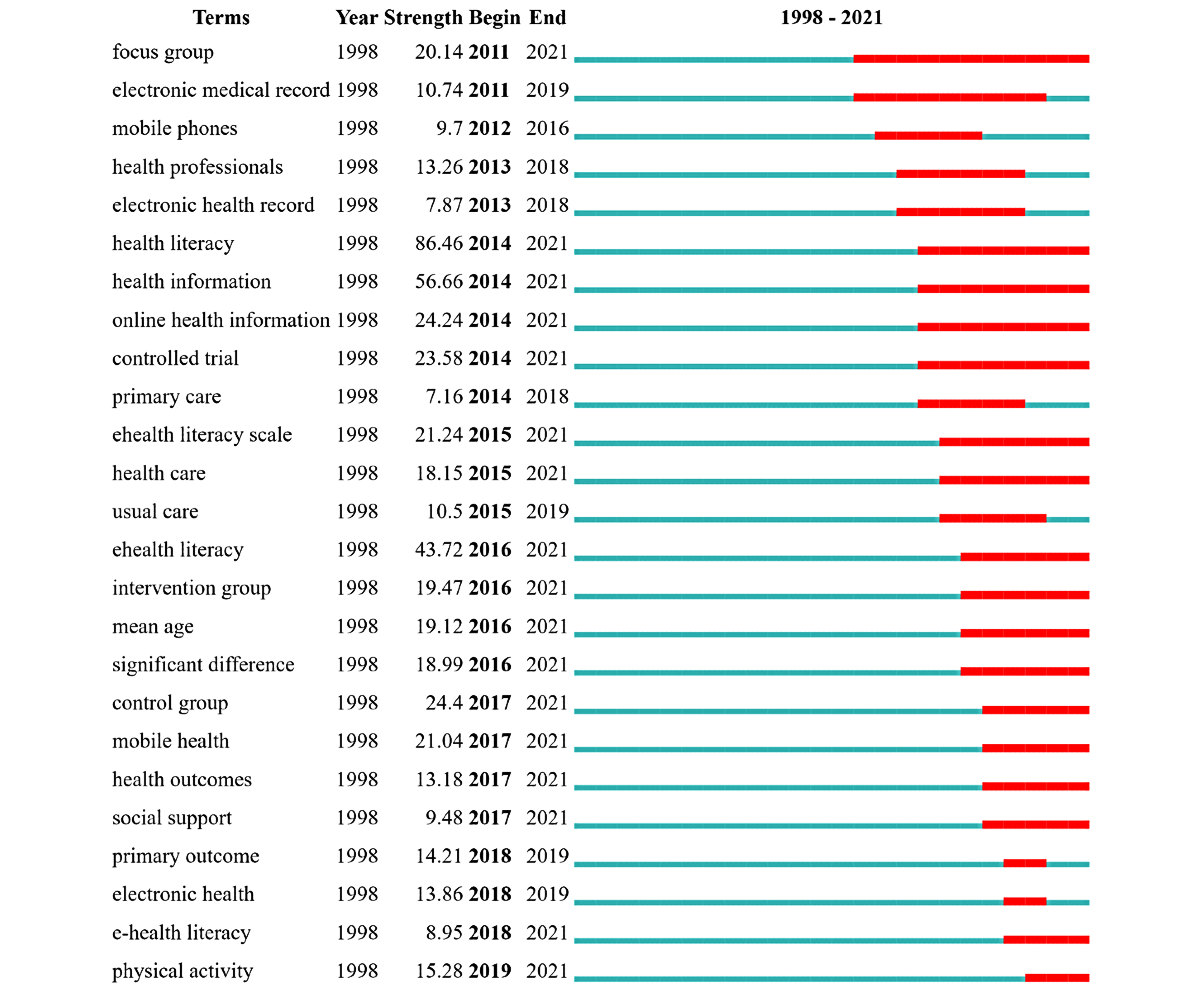
Top 25 terms with the strongest citation bursts.

To better understand the time trends of research topics, we used CiteSpace to analyze theme term bursts. The bursting of a term refers to when the citation strength of a term suddenly grows, which can reflect that the term has attracted more attention than before. The burst strength is calculated by the default Kleinberg algorithm of CiteSpace. The theme terms were extracted from the titles and abstracts of the citing articles. The top 20 terms in each year were selected to construct a co-occurrence network of theme terms. On this basis, the top 46 theme terms with strong burstiness were extracted, which were all the burst items found by CiteSpace. The list of these terms was ranked by the starting year of bursts. Figure 6 reports the top 25 strongest theme terms, and the list of all 46 theme terms citation bursts is reported in Multimedia Appendix 4. In Figure 6, “terms” in the first column refers to the theme terms, “year” in the second column refers to the year in which a theme term first occurred, “strength” refers to the citation bursts strength of theme terms, “begin” refers to the starting year in which a theme term burst, and “end” refers to the end year of a bursting theme term.

The theme terms related to the digital health literacy have the following features. Early studies mainly used theme terms that included “electronic medical record” (which was widely cited from 2011 to 2019) and “electronic health record” (which was widely cited from 2013 to 2018); this indicates that studies mainly focused on health records and health information before 2014, which was the gestation period of digital health literacy. The second burst peak appeared in 2014 with “health literacy,” and the strength of this burst continues today. In addition, “health information” and “online health information” also began to burst and grow in strength at this stage. Theme terms related to “health literacy” and “health information” at this stage have remained trending topics. Since 2015, the “eHealth Literacy Scale” has gained popularity. From 2016 to the present, theme terms such as “ehealth literacy” and “electronic literacy” have become popular. “eHealth literacy” has become a trending research topic. Thus, the concept of digital health literacy has gradually matured, and relevant research has exploded.

**Table 5:** Top 10 research categories.

WoS^a^ categories	Publications, n (%)
Health care sciences services	569 (29.1)
Medical informatics	436 (22.3)
Public environmental occupational health	427 (21.8)
Health policy services	143 (7.3)
Information science library science	127 (6.4)
Nursing	116 (5.9)
Medicine general internal	111 (5.6)
Oncology	78 (3.9)
Computer science information systems	76 (3.8)
Communication	69 (3.5)

^a^WoS: Web of Science.

### Co-occurrence of Keywords

**Figure 7:**
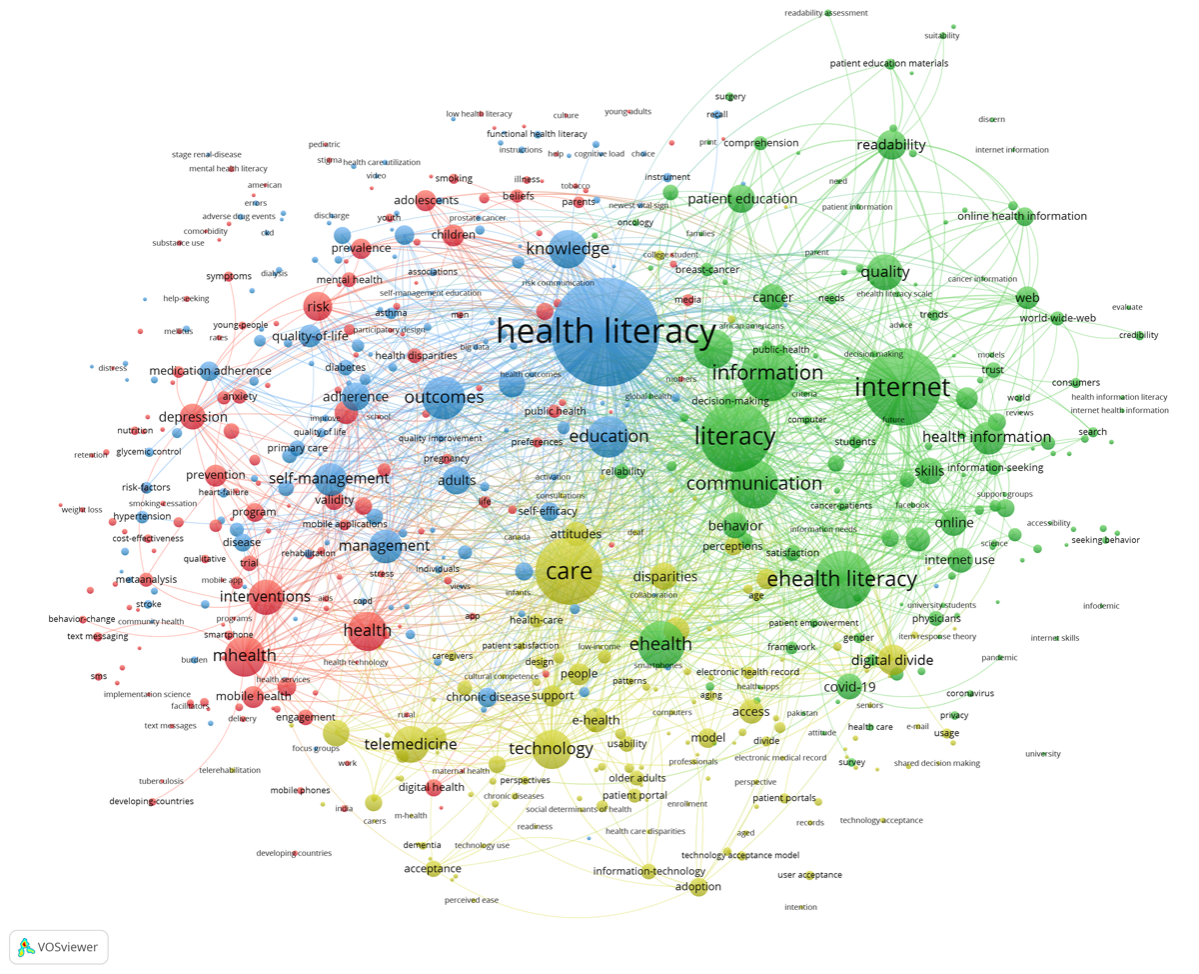
Keyword co-occurrence network.

The aforementioned research topics analysis obtained theme terms using the titles and abstracts of the surveyed articles. In this section, we analyze the co-occurrence network of keywords. Keywords can indirectly reveal trends and changes in research topics, which is crucial to understanding their development [36]. We used VOSviewer to construct a keyword co-occurrence network (Figure 7). Additionally, Table 6 shows the top 25 keywords according to occurrence. The most frequently occurring keyword was “health literacy,” which appears 666 times and has 580 links, with a total link strength of 4500. In addition, “internet,” “literacy,” “care,” and “eHealth literacy” are also frequently used keywords that rank highly in both occurrence frequency and connection strength.

**Figure 8:**
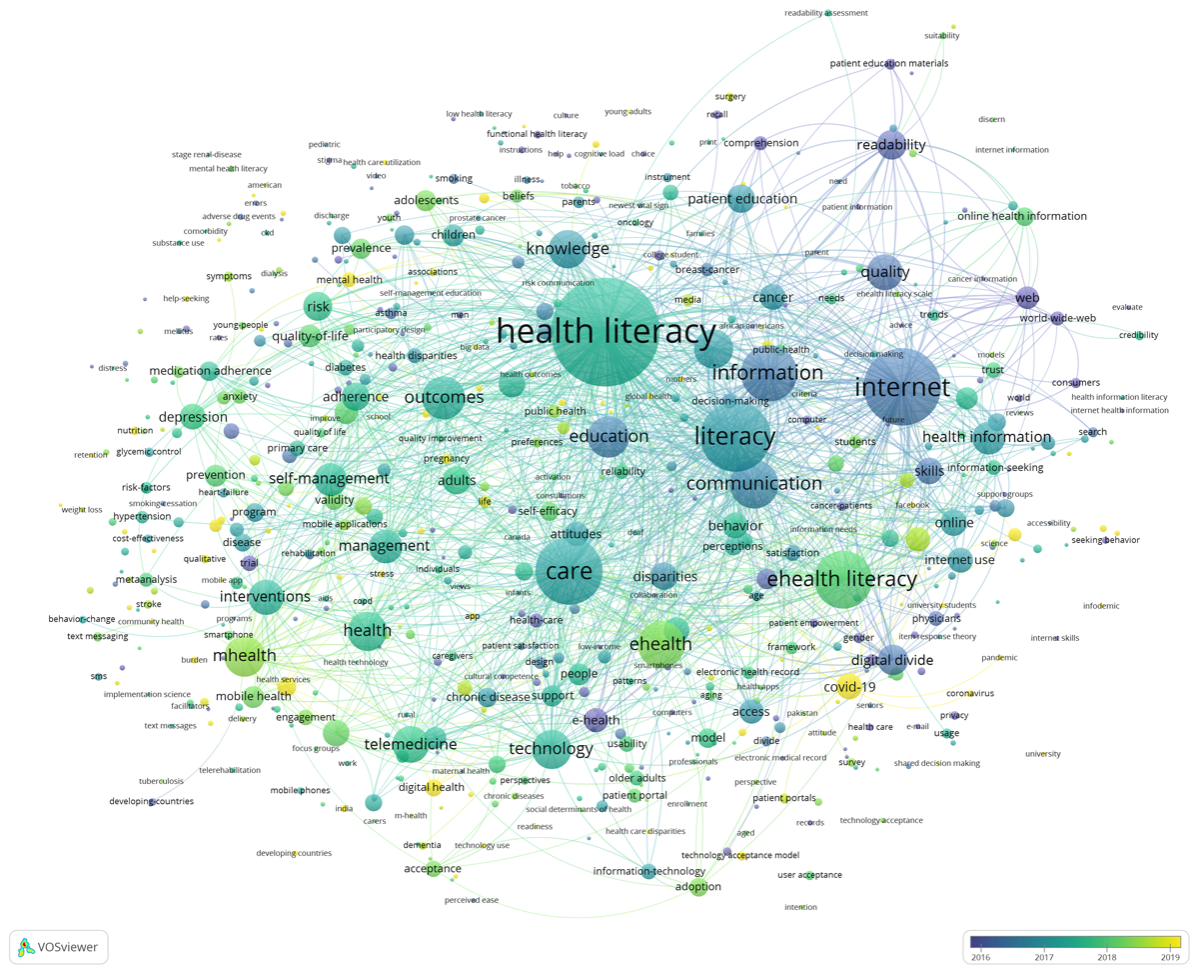
Overlay visualization of keyword co-occurrences.

To better elucidate the co-occurrence relationship between digital health literacy keywords and changing trends, VOSviewer was used to identify a total of 612 different keywords with 5 or more occurrences. Figures 7 and 8 represent the network and overlay visualization of keyword co-occurrence, respectively. There are 4 clusters in both figures. These clusters were organized by using the default VOS clustering, which is a clustering algorithm similar to modularity-based clustering. In Figure 7, color represents cluster, node size refers to frequency of appearance, and link thickness represents co-occurrence intensity (See Multimedia Appendix 6 for full-size image).

**Table 6:** Top 25 most frequently occurring keywords of digital health literacy.

Keyword	Occurrences^a^, n (%)	Links^b,c^, n (%)	Total link strength
Health literacy	666 (7.9)	580 (2.5)	4500
Internet	397 (4.7)	495 (2.1)	2973
Literacy	339 (4.0)	491 (2.1)	2587
Care	316 (3.7)	498 (2.2)	2416
eHealth literacy	252 (3.0)	422 (1.8)	1867
Information	241 (2.9)	432 (1.9)	1903
Communication	194 (2.3)	409 (1.8)	1625
eHealth	186 (2.2)	408 (1.7)	1443
Outcomes	159 (1.9)	374 (1.6)	1274
Education	152 (1.8)	355 (1.5)	1143
mHealth	148 (1.8)	346 (1.5)	1095
Health	141 (1.7)	340 (1.5)	955
Impact	139 (1.6)	361 (1.6)	1085
Technology	138 (1.6)	345 (1.5)	1153
Knowledge	134 (1.6)	330 (1.4)	1029
Telemedicine	127 (1.5)	306 (1.3)	875
Quality	122 (1.4)	292 (1.3)	926
Interventions	117 (1.4)	339 (1.5)	968
Management	110 (1.3)	317 (1.4)	897
Self-management	106 (1.3)	304 (1.3)	901
Health information	105 (1.2)	260 (1.1)	797
Digital divide	97 (1.2)	258 (1.1)	805
Readability	90 (1.1)	201 (0.9)	671
Risk	87 (1.0)	263 (1.1)	645
Patient education	86 (1.0)	227 (1.0)	631

^a^N=8434.

^b^N=23,124.

^c^Links refers to the number of keywords linked to a given keyword in the keyword co-occurrence network; total link strength refers to the total strength of the co-occurrence links of a given keyword with other keywords. The full list of co-occurrence keywords can be found in Multimedia Appendix 5.

As shown in Figure 7, the blue cluster mainly consists of health literacy keywords and the key factors affecting health literacy. The keywords include “health literacy” (n=666, 7.9%), “outcomes” (n=159, 1.9%), “education” (n=152, 1.8%), “knowledge” (n=134, 1.6%), and “self-management” (n=106, 1.3%). The core keywords that appear in the green cluster are “internet” (n=397, 4.7%), “literacy” (n=339, 4%), “eHealth literacy” (n=252, 3%), “information” (n=241, 2.9%), and “eHealth” (n=186, 2.2%). The co-occurrence intensity between these keywords is high, indicating that digital health literacy is closely related to internet and information literacy. The core keywords in the yellow cluster are “care” (n=316, 3.7%), “technology” (n=138, 1.6%), “telemedicine” (n=127, 1.5%), “digital divide” (n=97, 1.2%), “disparities” (n=81, 0.9%), “telehealth” (n=77, 0.9%), and “access” (n=67, 0.8%). These keywords form a co-occurrence network focused on the related technologies of care, the digital divide, disparities, and care access. The red cluster relates largely to digital health and has the core keywords “mHealth” (n=148, 1.8%), “health” (n=141, 1.7%), “interventions” (n=117, 1.4%), “risk” (n=87, 1%), and “depression” (n=74, 0.9%). These describe the detection of issues and health interventions as well as health risks using digital technology.

Figure 8 is the overlay visualization of keywords. In this figure, color represents average time of occurrence, node size represents occurrence frequencies, and link thickness represents co-occurrence strength (See Multimedia Appendix 7 for full-size image). Dark blue indicates an earlier appearance, and yellow indicates a more recent appearance. In terms of average time of occurrence, “information literacy” was the earliest keyword to appear, followed by “electronic health” and “mobile health,” which gradually changed to “digital health literacy” and expanded to include subfields like COVID-19 and mental health.

### Author Distribution

Table 7 presents the top 10 most productive authors. A total of 20 articles were published by Lyles, accounting for 1% of all 1955 articles. Lyles ranks first in terms of citations (n=386, 1.4%), with 19 links and a total link strength of 72, indicating that most articles were completed in collaboration with others. Wolf also published 20 (1%) articles. Schillinger published 17 articles (0.9%), ranking third for publications. Accordingly, we found that some of the most productive authors, namely, Wolf and Schillinger [25], were also the most productive in the field of health literacy. In addition, we investigated the h-index and found that Stellefson’s research may have had great impaction in the fields of digital health literacy. The i10-index and g-index can be used to complement the h-index.

**Figure 9:**
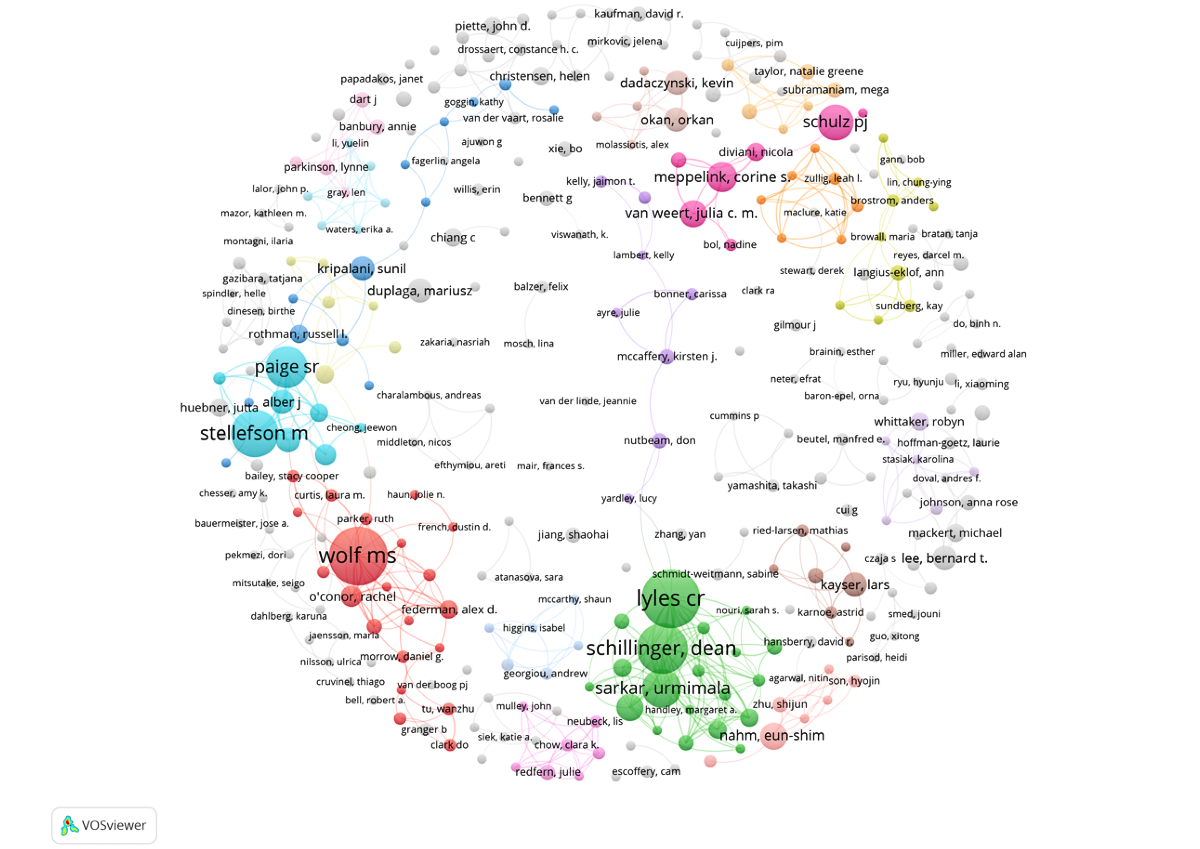
Coauthorship network based on publications.

We also analyzed coauthorships using VOSviewer. The coauthorship network comprises 323 authors who have published more than 3 articles each (Figure 9 ). In the figure, color represents cluster, node size represents publications, and link thickness represents collaboration strength.

There are 103 clusters in total. The 4 most significant clusters are green, red, azure, and magenta. The green cluster includes Lyles (20 publications, total link strength 72), Schillinger (17 publications, total link strength 65), and Ratanawongsa (9 publications, total link strength 34). The high total link strength among collaborative authors indicates a strong co-operative relationship. All 3 authors are colleagues at the University of California San Francisco. The article with the highest number of citations analyzes the obstacles to and promoting factors for the use of online portals by patients and caregivers. The study found that participants with limited health literacy faced more basic barriers, including reading and typing challenges, limited personal experience with online security vulnerabilities/viruses, and distrust in potential security measures [37]. Another study they collaborated on suggested that the health literacy of a patient population was a powerful indicator of which patients needed the most support in using health technologies [38].

In the red cluster, Wolf (20 publications, total link strength 49) represents the core node, accompanied by O'Conor (7 publications, total link strength 23) and Federman (6 publications, total link strength 22). Their research focused on how health literacy affects access to and use of health technologies [39,40]. They also summarized clinical [41] and randomized clinical trials [42] to explore the impact of health literacy differences on clinical outcomes.

**Table 7:** Top 10 most productive authors.

Author	Publications^a^, n (%)	Citations^b^, n (%)	Links	Total link strength	i10-index	G-index	H-index
Lyles	20 (1)	386 (1.4)	19	72	7	19	8
Wolf	20 (1)	285 (1.1)	16	49	7	16	8
Schillinger	17 (0.9)	266 (1)	16	65	5	16	7
Stellefson	16 (0.8)	466 (1.7)	7	50	10	16	10
Paige	14 (0.7)	452 (1.7)	7	47	9	14	9
Sarkar	13 (0.7)	340 (1.3)	14	42	6	13	8
Schulz	12 (0.6)	224 (0.8)	2	4	8	12	8
Meppelink	10 (0.5)	253 (0.9)	4	17	6	10	7
Van Weert	9 (0.5)	265 (1)	4	14	6	9	7
Ratanawongsa	9 (0.5)	210 (0.8)	11	34	3	9	5

^a^N=1955.

^b^N=27,012.

Stellefson (16 publications, total link strength 50) and Paige (14 publications, total link strength 47) form the core of the azure cluster. The co-operation intensity between them is high, and they are both from the University of Florida. Their research not only explores the factors that influence eHealth literacy and health information acquisition behavior [8,43] but also evaluates the reliability of eHEALS scores for patients with chronic illnesses [44]. They also examine literacy heterogeneity in the relationship between eHealth literacy and trust in online health communication channels and information sources [45] as well as test the measurement invariance of the eHEALS. Scholars believe that the eHEALS can be used to assess, monitor, and evaluate internet users' understanding of eHealth resources, information search skills, and participation abilities [46].

The magenta cluster includes Schulz (12 publications, total link strength 4), Meppelinks (10 publications, total link strength 17), and Van Weert (9 publications, total link strength 14). Schulz's co-operation strength is relatively low because most of his collaborators published fewer than 3 articles. He tested the measurement invariance of the eHEALS across countries [47] and examined the reliability and validity of the Italian version of the eHEALS. Schulz’s latest research analyzes the impact of subjective and objective health literacy on patients' judgment of health information and their decision-making ability [48].

Meppelinks and Van Weert, both from the University of Amsterdam, have a strong coauthorship relationship. Their articles analyze the impact of textual difficulty and illustrations on populations with low or high health literacy. They found that low health literacy groups benefit from well-illustrated information and nonchallenging texts, whereas high health literacy groups benefit from challenging texts [49]. They also analyzed the effectiveness of health animations among groups with differing health literacy. Narrated animations were revealed to be the best way to convey complex health information to people with low health literacy; this form can even bridge the information processing gap between low health literacy and high health literacy audiences [50]. Additionally, they explore the role of health literacy in the evaluation of online health information. Their study suggests that the differing evaluative abilities of people with different health literacies might be related to variances in issue perception and evaluation criteria [51]. They also evaluated the credibility, usefulness, and persuasiveness of both positive and negative texts on vaccination to examine the relationship between confirmation bias and health literacy in online health information searches. They determined that biased choices and biases against information persuasiveness were more prevalent among highly health-literate individuals, suggesting that bias recognition is important in the context of vaccination [52].

**Figure 10:**
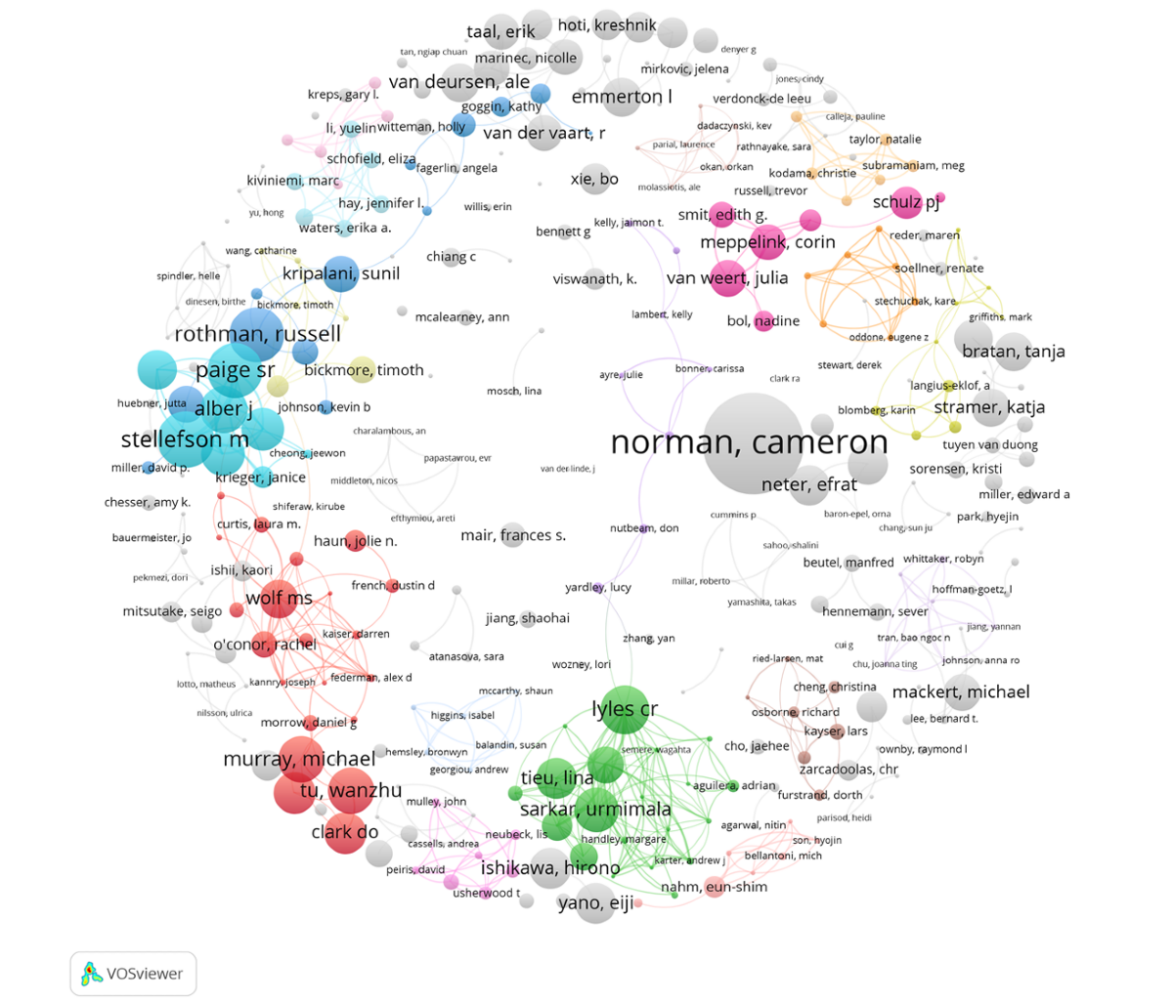
Coauthorship network of authors based on citations.

The number of articles published by an author does not reflect the quality or popularity of their work. We thus further analyzed the coauthorship network based on citations (Figure 10). Compared with Figure 9, each parameter in Figure 10 is the same except node size, which represents the number of citations. As shown in Figure 10, the 3 articles by Norman have been cited 995 times. His most notable contribution was the introduction of the concept of eHealth literacy in 2006. He defined eHealth literacy as the ability to seek, discover, understand, and evaluate health information from electronic sources and to apply the knowledge gained to solve health problems. As previously mentioned, his paper proposed the eHealth Literacy Scale, which includes 6 core literacies: traditional literacy, health literacy, information literacy, scientific literacy, media literacy, and computer literacy. This research was published in the *Journal of Medical Internet Research* and played a key role in the development of digital health literacy [8]. Another important paper by Norman detailed the design of the eHEALS, which not only evaluates consumers' perception of using information technology to promote health but also helps to determine whether eHealth programs match consumers’ needs [12]. The scale is widely used in follow-up studies on digital health literacy.

In addition, Rothman and Russell coauthored 6 publications with a total of 449 citations. Their study in collaboration with DeWalt found that primary care–based heart failure self-management programs can reduce the risk of hospitalization or death among low health-literate patients [53]. Another study by Rothman and Russell suggested that many parents do not understand the common health information needed to take care of an infant. In light of this, a new Parental Health Literacy Activities Test (PHLAT) was developed to evaluate parents' health literacy [54]. In addition, Rothman and Russell also evaluated the reliability and effectiveness of Brief Health Literacy Screen (BHLS) in routine clinical settings [55].

**Figure 11:**
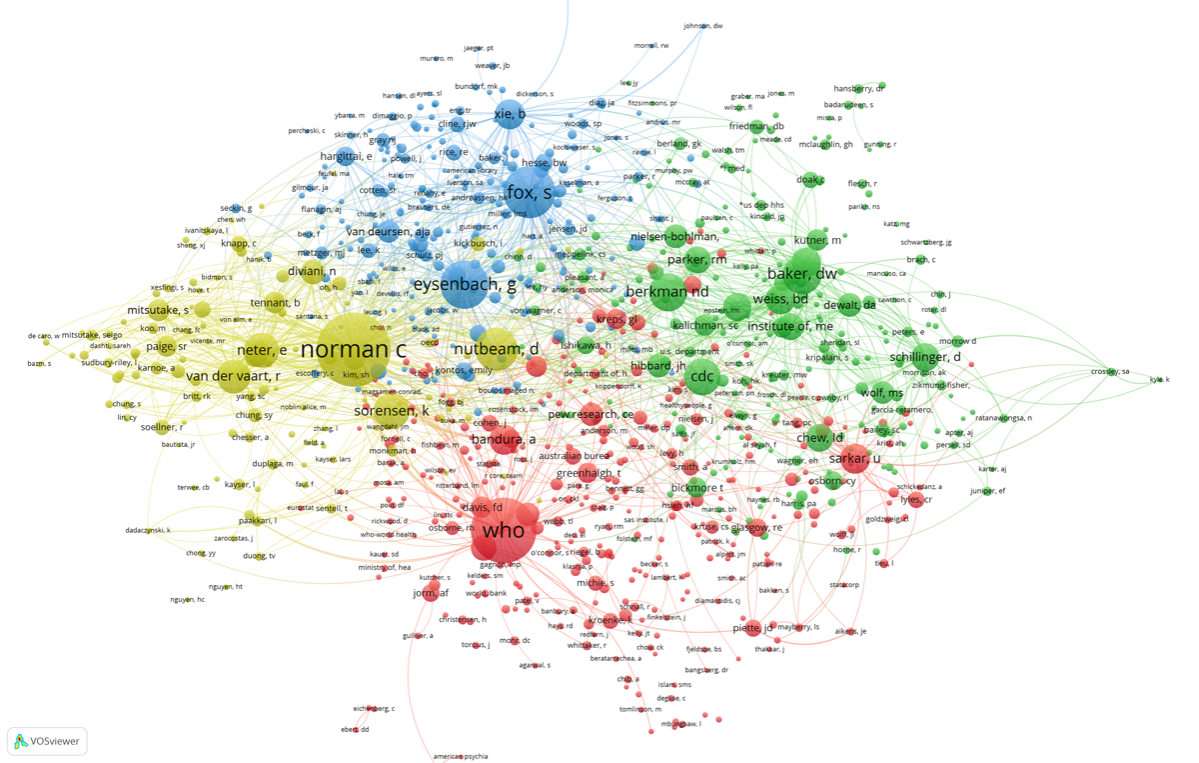
Co-citation network.

To better reflect the impact and contributions of the authors' research, we conducted a co-citation analysis of the cited references in 1955 articles. The co-citation network was established by selecting authors with more than 10 citations. A total of 919 authors appeared in the network (selected from a total of 40,628), forming the 4 clusters shown in Figure 11. In this figure, color represents cluster, node size represents citations, and link thickness represents co-citation strength (see Multimedia Appendix 8 for full-size image).

Figure 11 shows that Norman represents the core node in the yellow cluster. Norman had the most citations and strongest co-citation strength. As previously mentioned, he introduced the electronic health literacy model [8] and designed the eHEALS [12] and thus played a very important role in the development of digital health literacy research. Another notable author is Nutbeam, who conducted various research on many aspects of digital health literacy, such as eHealth using with low levels of health literacy [56,57], attitudes of people with different health literacy toward digital health interventions, and skills for telehealth [58].

The WHO is the key node in the red cluster. As an agency author, the WHO has always played a pivotal role in the health practice field, including digital health literacy.

Fox is the most prominent author in the blue cluster. She worked on the Pew Research Center’s internet project from 2000 to 2014 and mainly studied the impact of internet technology on health. During her time at the Pew Research Center, she published several highly cited research reports on health information [59], mobile health [60], health online [61], and other topics. From 2015 to 2017, she was the chief technology officer of the US Department of Health and Human Services. Another notable author in the blue cluster is Eysenbach, who made significant contributions to the definition of eHealth [62] and the evaluation of internet health information quality [63,64].

Baker, the most significant author in the green cluster, developed a simple scale measuring functional health literacy early in 1999 [65]. In 2006, he designed a concept model of health literacy that received widespread attention and played an important role in promoting the definition and measurement of health literacy [66]. Berkman is also an important author in the green cluster. He contributed to the definition of health literacy [67] and proposed that low health literacy is related to poor health outcomes and poor use of health care services [20]. The Centers for Disease Control and Prevention (CDC) are also in the green cluster, indicating that this agency has paid great attention to digital health literacy.

### Articles With High Citation Numbers

Based on the keyword co-occurrence and author co-operation analysis, we further explored the cited articles. Table 8 reports the top 10 articles according to number of citations. We concluded that these 10 articles focus on 4 research themes: definition and measurement of digital health literacy, digital health literacy and health outcomes, digital health literacy and the digital divide, and the influencing factors of digital health literacy.

Two of Norman's articles are the most prominent with regard to definition and measurement of digital health literacy. As noted, the eHealth literacy conceptual model [8] and eHEALS [12] have contributed significantly to the study of eHealth literacy. Ishikawa et al [71] proposed and examined a newly developed scale that measures 3 different aspects of health literacy (ie, functional, communicative, and critical). This scale is a reliable and effective measurement method for the 3 types of health literacy in diabetic patients. Exploring patients’ health literacy may lead to a better understanding of potential barriers to patients’ self-management and health promoting behaviors [71].

Additionally, 3 articles discussed the theme of digital health literacy and health outcomes. Baker found that insufficient functional health literacy increases hospitalization risk [68]. Murray conducted a randomized clinical trial from a pharmacist intervention perspective and followed the trial with an electronic monitor, finding that patient health literacy significantly influenced the impact of pharmacist interventions on health outcomes [69]. DeWalt et al [53] determined that a primary care–based heart failure self-management program designed for low-literacy patients reduced risk of hospitalization or death.

Moreover, 2 articles analyzed the theme of digital health literacy and the digital divide. Neter and Brainin [22] explored the digital divide in relation to health information and argue that the internet reinforces existing social disparities. They maintain that a more comprehensive and sophisticated use of the internet alongside the growth of a highly eHealth–literate population has created new inequalities in the digital health information arena. Focusing on the digital divide among low-income home-based seniors, Choi and DiNitto surveyed internet use patterns, reasons for stopping use, eHealth literacy, and attitudes toward computer/internet use among low-income home-based seniors and younger adults under the age of 60. The study found that internet usage among this population is very low compared to the overall US population base due to either lack of access to computers and internet technologies, lack of financial resources to obtain computers and access technologies for personal use, or limitations due to medical conditions and disabilities [70].

Furthermore, 2 articles studied the influencing factors for digital health literacy. As mentioned earlier, Tennant et al [43] explored the extent to which sociodemographics, social determinants, and electronic device use influence eHealth literacy and the use of Web 2.0 for health information among baby boomers and older adults. Their study found that higher eHealth literacy among baby boomers and the older adults was related to being younger and more educated. Using a HealthSpace case study, Greenhalgh et al [72] found that policymakers hoped for patient empowerment, personalized care, lower health care costs, better data quality, and improved health literacy through personal electronic health records. However, these records are not being used adequately. Because personal electronic health records must be closely aligned with people's attitudes, self-management practices, identified information needs, and health care options, the risk of them being abandoned or not adopted at all is significant.

**Table 8:** Top 10 articles according to number of citations.

Title	Author	Journal	Year	Citations	Reference	Theme
“eHealth literacy: Essential skills for consumer health in a networked world”	Norman and Skinner	*Journal of Medical Internet Research*	2006	722	[8]	Definition and measurement of digital health literacy
“Health literacy and the risk of hospital admission”	Baker et al	*Journal of General Internal Medicine*	1998	501	[68]	Digital health literacy and health outcomes
“Pharmacist intervention to improve medication adherence in heart failure - A randomized trial”	Murray et al	*Annals of Internal Medicine*	2007	297	[69]	Digital health literacy and health outcomes
“eHealth literacy: extending the digital divide to the realm of health information”	Neter and Brainin	*Journal of Medical Internet Research*	2012	282	[22]	Digital health literacy and the digital divide
“eHEALS: The eHealth Literacy Scale”	Norman and Skinner	*Journal of Medical Internet Research*	2006	271	[12]	Definition and measurement of digital health literacy
“eHealth Literacy and web 2.0 health information seeking behaviors among baby boomers and older adults”	Tennant et al	*Journal of Medical Internet Research*	2015	253	[43]	Influencing factors for digital health literacy
“The digital divide among low-income homebound older adults: internet use patterns, eHealth literacy, and attitudes toward computer/internet use”	Choi and DiNitto	*Journal of Medical Internet Research*	2013	248	[70]	Digital health literacy and the digital divide
“Measuring functional, communicative, and critical health literacy among diabetic patients”	Ishikawa et al	*Diabetes Care*	2008	220	[71]	Definition and measurement of digital health literacy
“A heart failure self-management program for patients of all literacy levels: A randomized, controlled trial” [ISRCTN11535170]	DeWalt et al	*BMC Health Services Research*	2006	213	[53]	Digital health literacy and health outcomes
“Adoption, non-adoption, and abandonment of a personal electronic health record: case study of HealthSpace”	Greenhalgh et al	*BMJ-British Medical Journal*	2010	174	[72]	Influencing factors for digital health literacy

### Co-cited Literature

**Figure 12:**
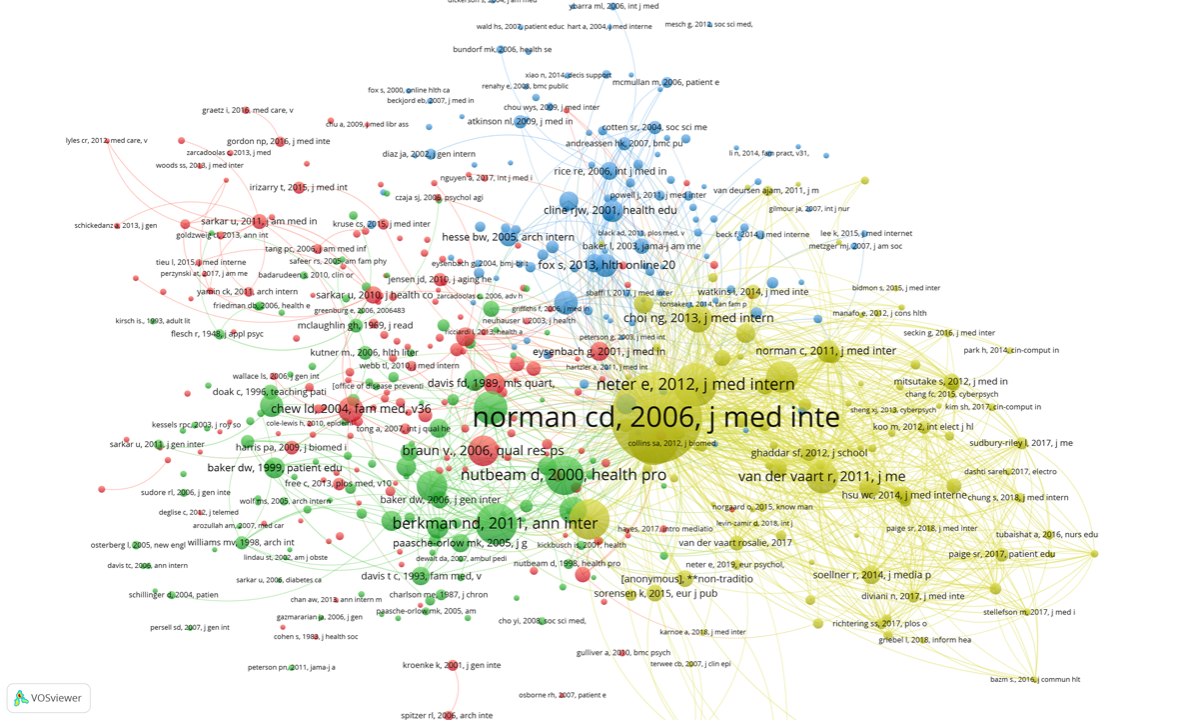
Co-citation network of cited references.

To further examine literature that has played an important role in promoting the field of digital health literacy, we also conducted co-citation network analysis of references cited by the 1955 articles. A total of 510 references with more than 10 citations frequencies were selected as the co-citation network nodes, and 4 clusters were formed, as shown in Figure 12. In this figure, color represents cluster, node size represents citations, and link thickness represents co-citation strength. The full-size figure can be found in Multimedia Appendix 9.

The articles in the yellow cluster focus on the definition and scale of digital health literacy. Norman and Skinner [8,12] are at the core of the whole co-citation network. Within the yellow cluster, the article by Neter and Brainin [22] extends the digital divide to health information and health inequalities. Sørensen’s [73] article promotes the development of definitions and conceptual models for health literacy. His research proposes a model integrating public views of medicine and health literacy. The model can be used as a conceptual basis for the development of interventions to improve health literacy and the development and validation of health literacy measurement tools. In addition, the paper tests by van der Vaart, the eHEALS by Norman, and the Dutch version of the eHEALS can be used to test for sufficient internal consistency and predictive validity [74].

In Figure 12, articles shown in the green cluster have supported research on health literacy and health outcomes. Berkman [20] proposed that low health literacy is related to poor health outcomes and poor use of health care services. Nutbeam [75] proposed a health outcomes model emphasizing health literacy an important result of health education. Nutbeam’s study of health literacy as a concept showed the differences between functional, interactive, and critical health literacy and determined that improving health literacy entails not only transmitting information but also developing skills such as reading booklets and making successful appointments.

The red cluster includes articles that establish the theoretical basis and research methods of digital health literacy research. For example, Braun [76] advocates for thematic analysis as a useful and flexible method for qualitative psychological research. The testing scale of perceived usefulness and perceived ease of use developed by Davis [77] is very important and provides basic support for research on user acceptance. Digital health literacy often involves issues such as measuring users' acceptance of digital technology, and Davis’ study has therefore been widely cited. A study by Chew [78] discussed the simple problem of identifying patients with poor health literacy and presented the Short Test of Functional Health Literacy in Adults (STOHFLA) scale, which has 3 key screening questions: ”How often can someone help you read hospital information?“ ”How confident are you in filling out your own medical forms?” and “How often do you have questions about your health due to difficulty understanding written information?” These 3 questions effectively screen for health literacy inequality [78]. Hence, Chew’s study provided a basis for later measurement of and scale construction for digital health literacy.

Research in the blue cluster focuses on health literacy and the digital divide. Cline [79] carried out a literature review and explored consumers' search for health information on the internet. Her paper discussed the criteria for evaluating online health information and argues that attention should be paid not only to “network gap” and information quality but also to the communication and transaction quality inherent in internet use [79]. Kontos [80] examined the use of eHealth tools according to sociodemographic factors such as race/ethnicity, socioeconomic status, age, and gender and found that there is a digital divide—compared with their peers, adults with poor economic conditions, adults who are older, and males are less likely to participate in a large number of eHealth activities [80].

**Figure 13:**
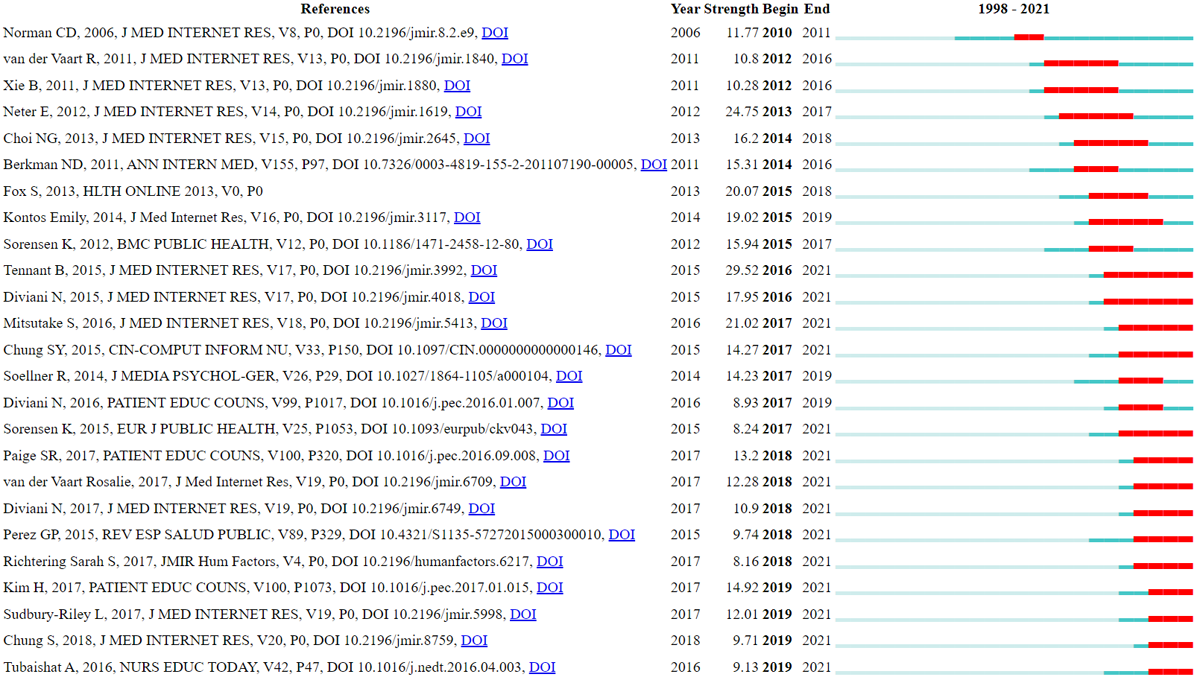
Top 25 references with the strongest citation bursts among a co-citation network of cited references.

The cited references include strong citation bursts reflecting changes in trending topics over time. A citation burst indicate the citations of an article increase rapidly within a period time. In this study, CiteSpace was used to analyze bursts of co-cited articles. The co-citation network was constructed based on the total number of the top 20 cited references each citation year. There were 1830 nodes and 6236 links. The 59 burst items found by CiteSpace are reported in Multimedia Appendix 10. Figure 13 reports the top 25 articles of 59 burst references with the strongest burstiness. The references are ranked by the starting year of the burst. In Figure 13, “references” in the first column refers to the list of publications with high burst; “year” in the second column refers to the year in which a given publication was first cited; “strength” refers to the citation burst strength of a given publication, which was calculated by the default Kleinberg algorithm of CiteSpace; “begin” refers to the year in which the citation of a publication begins to burst; and “end” refers to the end year of a bursting publication.

The article that burst earliest is that of Norman and Skinner [8]. In 2021, 2 articles burst, namely, those of van der Vaart et al [74] and Xie [81]. Van der Vaart et al [74] examined the validity of the Dutch version of the eHEALS, while Xie [81] tested the effect of electronic health literacy intervention for the elderly. Regardless of the specific learning method employed, the tested electronic health literacy intervention significantly improved the efficacy of digital health literacy and led to positive changes in self-managed health care [81]. Neter and Brainin burst suddenly from 2013 to 2017 and had a strong burstiness of 24.75 [22]. The burstiness of Tennant et al [43] was the highest among all cited articles. Their citations significantly burst from 2016 until 2021. In addition, the article published by Diviani in 2015 [82] burst at the same stage from 2016 to 2021.

In 2017, 5 articles were frequently cited. In 1 of these, Mitsutake [83] studied the relationship between eHealth literacy and the healthy behaviors of adult internet users. The study found that some healthy behaviors, including exercise and balanced nutrition, are independently related to eHealth literacy in Japan [83]. Citations of this article in 2017 burst and lasted until 2021, with a high bursting strength of 20.39.

In 2018, 4 articles had the strongest citation bursts, which lasted until 2021. Paige [44] assessed the reliability of the eHEALS for patients with chronic diseases. Van der Vaart [6] argued that previous tools for measuring digital health literacy focused on information collection (Health 1.0 skills) but not on network interactions (Health 2.0) and therefore developed the new Digital Health Literacy Instrument (DHLI) to measure operating skills, navigation skills, information searching, evaluation of reliability, determining relevance, adding self-generated content, and protecting privacy [6]. The burst duration of this article lasted from 2018 to 2021. Diviani [13] examined the reliability and validity of the Italian version of the eHEALS (I-eHEALS). Perez [14] validated the Spanish version of the eHEALS.

Of the 4 articles that burst in 2019, 1 was a literature review, while the other 3 were extensions of the eHEALS study. Kim [84] conducted a literature review on health literacy in the electronic age. Sudbury-Riley [47] tested the multinational test invariance of the eHEALS; Chung [16] developed the Korean version of the eHEALS; and Tubaishat [85] studied electronic health literacy among nursing students.

## Discussion

### Principal Findings

In this study, we conducted a comprehensive bibliometric analysis of the field of digital health literacy. A total of 1955 scientific publications were retrieved from the WoS Core Collection, and the development of digital health literacy over the past 20 years was analyzed. This paper systematically summarizes the development context and trends of digital health literacy research, and our work serves as a basic reference and directional guide for future research in this field. Our study uncovered the most productive countries, institutions, journals, and authors. It also identified the different stages of research trends in digital health literacy, the most important research themes of digital health literacy, and the evolution of the concept of digital health literacy.

Our analysis of national and regional contributions revealed that the United States is the leader in this field in terms of both publications and citations. In relation to institutional contributions, the University of California System is in the top position. Our analysis of institutional collaboration networks shows that the focus on digital health literacy research is gradually expanding from high-income countries to emerging market countries and regions. The *Journal of Medical Internet Research* is the leading journal in digital health literacy research for number of articles, citations, and co-citations. In terms of author contributions, Lyles, Wolf, and Schillinger are the top 3 authors, while Norman has the highest number of citations. Among them, Wolf and Schillinger are also the most productive in the area of health literacy [25]. Regarding the co-citation of authors, Norman is the most co-cited individual author, and the WHO is the most co-cited institutional author.

Research on digital health literacy spans from 1998 to 2021. In terms of annual publications, the research history can be divided into 3 phases: (1) 1998 to 2005 was the incubation period, with almost no growth in the number of publications and the number of publications remaining in the single digits; (2) 2006 to 2013 was the slow growth period, with the number of publications remaining below 100; (3) after 2014, digital health literacy research entered a period of rapid growth, with the annual number of articles exceeding 100.

Thematic analysis demonstrated that health care sciences services, medical informatics, and public environmental occupational health are the 3 most important research fields related to digital health literacy. The theme bursting analysis revealed that early studies mainly focused on electronic medical records (widely cited from 2011 to 2019) and electronic health records (widely cited from 2013 to 2018), indicating that studies began to focus on topics such as health records and health information before 2014; this was also the gestation period for the concept of digital health literacy. The second bursting peak occurred in 2014 and has continued into the present. The theme terms related to health literacy and health information in this stage have become trending topics. In 2015, the eHEALS began to burst. Subsequently, thematic terms such as eHealth literacy began to emerge and trend. After 2014, the concept of digital health literacy gradually matured, and relevant studies began to explode. From the evolution of the theme’s citation bursts, concepts related to digital health literacy evolved from electronic medical record, electronic health record, eHealth Literacy Scale, to eHealth literacy. This evolution can be attributed to the advancement of digital technology and the digital health industry. In the early days, the use of electronic technology only turned paper health records into electronic health information, such as electronic medical records and electronic health records. With the escalating empowerment of the health industry by digital technology, especially the emergence of professional measurement tools such as the eHealth Literacy Scale, a moniker related to digital health literacy has gradually emerged. The emergence and evolution of this kind of terminology are in line with the general pattern of an emerging field of study from initiation to maturity.

The co-occurrence analysis of keywords yielded 4 clusters: keywords of health literacy and critical factors affecting health literacy; keywords of digital health literacy related to the internet and information literacy; keywords of related technologies to digital health literacy, the digital divide, disparities, and availability of care; and keywords related to the application of digital health. Based on the average frequencies of occurrence of keywords, information literacy was the earliest, followed by electronic health, mobile health, and digital health literacy, which expanded to digital health literacy niche research areas such as COVID-19 and mental health.

Our analysis of articles with high citations showed that they mainly focus on 4 aspects of digital health literacy: the definition and scale of digital health literacy [12], digital health literacy and health outcomes [53], digital health literacy and the digital divide [22,23], and influencing factors of digital health literacy [43]. Reference co-citation analysis revealed 4 clusters as well as the theoretical basis and research methods of digital health literacy research [77].

### Comparisons With Prior Work

Some scholars have conducted bibliometric analysis on health literacy and eHealth literacy. Among them, a few suggest that mental health literacy and eHealth literacy will be 2 expansion directions for future health literacy research [25]. In contrast to previous bibliometric articles in related fields, our study is a comprehensive bibliometric analysis of the field of digital health literacy, which includes studies on eHealth literacy. There are also early publications that conducted bibliometric analyses on internet health information–seeking behaviors [86] but did not further explore the relationship between this behavior and health literacy. Some scholars also conducted a bibliometric analysis of consumer health informatics (CHIN) and found that research topics focused on patient education, health information demands, health information search behavior, health behavior interventions, health literacy, health information technology, and eHealth [87]. However, health informatics is broader in scope, and our study focuses more on the emerging subfield of digital health literacy. In addition, a recent bibliometric analysis of global eHealth found that one of the frontier issues in global eHealth research is the eHealth Literacy Scale [26]. This coincides with the research on digital health literacy measurement tools summarized in this paper. Some scholars likewise used bibliometric methods to analyze research hotspots and trends in eHealth literacy, arguing that eHealth literacy research faces challenges such as the development of terminological connotations, the objectivity of assessment methodology, and the impact of interventions [27]. However, we extended eHealth literacy to a broader scope: digital health literacy. In addition to encompassing eHealth literacy, we further expanded the scope of the literature retrieval by using the intersection of digital technology and health literacy and the intersection of digital health and literacy. Therefore, this study provides a systematic analysis for the development of research on digital health literacy.

### Limitations

There are 3 main limitations of this study. First, in terms of data selection, the WoS core collection was selected, while other databases such as Scopus were not included. This study mainly focused on journals, and less attention was paid to other means of scientific knowledge dissemination (such as books, working papers, and reports). Therefore, some important studies may have been missed, especially emerging research.

Second, there are some subjective aspects in the sample selection in this paper. For example, on the one hand, we only analyzed English publications; thus, there may have been some linguistic bias. Future comparisons for articles published in different language or countries can be made. On the other hand, subjectivity may have influenced our search strategies and screenings as this is difficult to avoid in bibliometric studies.

Third, our study focused on a bibliometric analysis aimed at analyzing the structure of knowledge in the field of digital health literacy. There was no detailed discussion of study content. This calls for a more systematic literature review in the future.

### Future Directions

The aforementioned results and discussion reveal that there are many issues in the field of digital health literacy that deserve further study. In particular, considering the ongoing COVID-19 pandemic, digital health literacy deserves more attention from society and scholars. For example, it is important to consider how the government can promote health outcomes by enhancing the digital health literacy of the public to enhance COVID-19 prevention and control; how the digital health literacy of different groups can be measured to achieve effective COVID-19 prevention and control; and how the digital divide can be bridged by enhancing the digital health literacy of vulnerable groups to mitigate any health inequities caused by COVID-19.

## Appendix

Multimedia Appendix 1 Full-size Figure 3.

Multimedia Appendix 2 Full-size Figure 4.

Multimedia Appendix 3 Full-size Figure 5.

Multimedia Appendix 4 Theme terms citation burst for Figure 6.

Multimedia Appendix 5 Top 100 co-occurrence of keywords.

Multimedia Appendix 6 Full-size Figure 7.

Multimedia Appendix 7 Full-size Figure 8.

Multimedia Appendix 8 Full-size Figure 11.

Multimedia Appendix 9 Full-size Figure 12.

Multimedia Appendix 10 References citation burst for Figure 13.

## Abbreviations

A&HCI: Art and Humanities Citation Index
BHLS: Brief Health Literacy Screen
CDC: Centers for Disease Control and Prevention
CHIN: consumer health informatics
DHLI: Digital Health Literacy Instrument
eHEALS: eHealth Literacy Scale
FDA: Food and Drug Administration
mHealth: mobile health
PHLAT: Parental Health Literacy Activities Test
PLOS: Public Library of Science
SCIE: Science Citation Index Extension
SSCI: Social Science Citation Index
STOHFLA: Short Test of Functional Health Literacy in Adults
WHO: World Health Organization
WoS: Web of Science
