# Supplementary material for: Digital Health Literacy: Bibliometric Analysis
Source: J Med Internet Res. 2022 Jul 6;24(7):e35816. doi: 10.2196/35816 (PMC9301558; doi:10.2196/35816)
Supplement: Multimedia Appendix 4 [file jmir_v24i7e35816_app4.pdf]

Top 46 Terms with the Strongest Citation Bursts

Terms	Year	Strength	Begin	End	1998 - 2021
focus group	1998	20.17	**2011**	2021	
electronic medical record	1998	10.76	**2011**	2019	
health education	1998	7.06	**2011**	2016	
internet access	1998	6.7	**2011**	2014	
health behaviors	1998	5.11	**2011**	2017	
digital divide	1998	4.46	**2011**	2013	
mobile phones	1998	9.7	**2012**	2016	
health care providers	1998	6.88	**2012**	2017	
adequate health literacy	1998	6.78	**2012**	2015	
descriptive statistics	1998	3.68	**2012**	2014	
practice implications	1998	3.54	**2012**	2013	
confidence interval	1998	3.52	**2012**	2016	
functional health literacy	1998	3.52	**2012**	2014	
health professionals	1998	13.26	**2013**	2018	
electronic health record	1998	7.88	**2013**	2018	
patient portals	1998	5.15	**2013**	2017	
mobile technology	1998	4.64	**2013**	2017	
social network	1998	4.12	**2013**	2017	
healthcare system	1998	3.97	**2013**	2016	
data collection	1998	3.44	**2013**	2015	
health literacy	1998	86.67	**2014**	2021	
health information	1998	56.81	**2014**	2021	
online health information	1998	24.29	**2014**	2021	
controlled trial	1998	23.62	**2014**	2021	
primary care	1998	7.17	**2014**	2018	
chronic condition	1998	6.92	**2014**	2017	
ehealth literacy scale	1998	21.27	**2015**	2021	
health care	1998	18.19	**2015**	2021	
usual care	1998	10.51	**2015**	2019	
internet user	1998	5.9	**2015**	2016	
health care system	1998	5.29	**2015**	2017	
patient education	1998	5.12	**2015**	2018	
ehealth literacy	1998	43.79	**2016**	2021	
intervention group	1998	19.5	**2016**	2021	
mean age	1998	19.14	**2016**	2021	
significant difference	1998	19.02	**2016**	2021	
limited health literacy	1998	6.93	**2016**	2017	
control group	1998	24.42	**2017**	2021	
mobile health	1998	21.05	**2017**	2021	
health outcomes	1998	13.21	**2017**	2021	
social support	1998	9.49	**2017**	2021	
primary outcome	1998	14.21	**2018**	2019	
electronic health	1998	13.87	**2018**	2019	
e-health literacy	1998	8.96	**2018**	2021	
physical activity	1998	15.29	**2019**	2021	
odds ratio	1998	6.86	**2019**	2021	
