# Supplementary figures and images for: Digital Health Literacy: Bibliometric Analysis
Source: J Med Internet Res. 2022 Jul 6;24(7):e35816. doi: 10.2196/35816 (PMC9301558; doi:10.2196/35816)

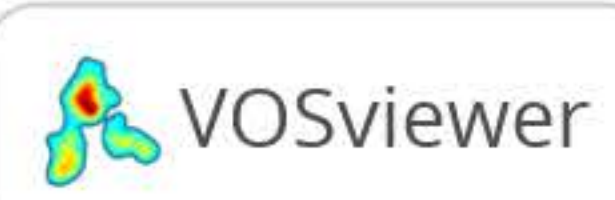

Supplement: Multimedia Appendix 6 [file jmir_v24i7e35816_app6.pdf]

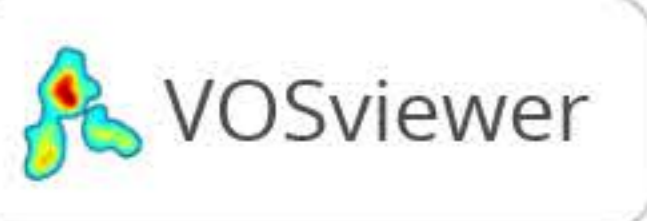

Supplement: Multimedia Appendix 8 [file jmir_v24i7e35816_app8.pdf]
