# Supplementary material for: Digital Health Literacy: Bibliometric Analysis
Source: J Med Internet Res. 2022 Jul 6;24(7):e35816. doi: 10.2196/35816 (PMC9301558; doi:10.2196/35816)
Supplement: Multimedia Appendix 10 [file jmir_v24i7e35816_app10.pdf]

Top 59 References with the Strongest Citation Bursts

References	Year	Strength	Begin	End	1998 - 2021
Paasche-Orlow MK, 2005, AM J RESP CRIT CARE, V172, P980, DOI 10.1164/rccm.200409-1291OC, DOI	2005	3.49	**2009**	2010	
Norman CD, 2006, J MED INTERNET RES, V8, P0, DOI 10.2196/jmir.8.2.e9, DOI	2006	11.77	**2010**	2011	
Nutbeam D, 2008, SOC SCI MED, V67, P2072, DOI 10.1016/j.socscimed.2008.09.050, DOI	2008	5.57	**2010**	2013	
Bodie Graham D, 2008, Health Mark Q, V25, P175, DOI 10.1080/07359680802126301, DOI	2008	4.45	**2010**	2013	
Kutner M, 2006, 2006483 NCES US DEP, V0, P0	2006	4.41	**2010**	2011	
Fox S, 2009, SOCIAL LIFE HLTH INF, V0, P0	2009	4.62	**2011**	2013	
Xie B, 2009, J HEALTH COMMUN, V14, P510, DOI 10.1080/10810730903089614, DOI	2009	3.73	**2011**	2012	
Ishikawa H, 2008, DIABETES CARE, V31, P874, DOI 10.2337/dc07-1932, DOI	2008	3.65	**2011**	2012	
van der Vaart R, 2011, J MED INTERNET RES, V13, P0, DOI 10.2196/jmir.1840, DOI	2011	10.8	**2012**	2016	
Xie B, 2011, J MED INTERNET RES, V13, P0, DOI 10.2196/jmir.1880, DOI	2011	10.28	**2012**	2016	
Stellefson M, 2011, J MED INTERNET RES, V13, P0, DOI 10.2196/jmir.1703, DOI	2011	7.7	**2012**	2016	
Berkman ND, 2010, J HEALTH COMMUN, V15, P9, DOI 10.1080/10810730.2010.499985, DOI	2010	7.24	**2012**	2015	
Chan CV, 2011, J MED INTERNET RES, V13, P0, DOI 10.2196/jmir.1750, DOI	2011	7.18	**2012**	2016	
Knapp C, 2011, J MED INTERNET RES, V13, P0, DOI 10.2196/jmir.1697, DOI	2011	5.63	**2012**	2016	
Norman C, 2011, J MED INTERNET RES, V13, P0, DOI 10.2196/jmir.2035, DOI	2011	4.79	**2012**	2014	
Neter E, 2012, J MED INTERNET RES, V14, P0, DOI 10.2196/jmir.1619, DOI	2012	24.75	**2013**	2017	
Sarkar U, 2011, J AM MED INFORM ASSN, V18, P318	2011	8.14	**2013**	2016	
Kreps GL, 2010, PATIENT EDUC COUNS, V78, P329, DOI 10.1016/j.pec.2010.01.013, DOI	2010	5.65	**2013**	2014	
Sarkar U, 2010, J HEALTH COMMUN, V15, P183, DOI 10.1080/10810730.2010.499988, DOI	2010	5.27	**2013**	2015	
Goel MS, 2011, J GEN INTERN MED, V26, P1112, DOI 10.1007/s11606-011-1728-3, DOI	2011	4.68	**2013**	2015	
Schulz PJ, 2013, PATIENT EDUC COUNS, V90, P4, DOI 10.1016/j.pec.2012.09.006, DOI	2013	3.79	**2013**	2016	
Choi NG, 2013, J MED INTERNET RES, V15, P0, DOI 10.2196/jmir.2645, DOI	2013	16.2	**2014**	2018	
Berkman ND, 2011, ANN INTERN MED, V155, P97, DOI 10.7326/0003-4819-155-2-201107190-00005, DOI	2011	15.31	**2014**	2016	
Archer N, 2011, J AM MED INFORM ASSN, V18, P515, DOI 10.1136/amiajnl-2011-000105, DOI	2011	4.4	**2014**	2015	
Osborne RH, 2013, BMC PUBLIC HEALTH, V13, P0, DOI 10.1186/1471-2458-13-658, DOI	2013	4.25	**2014**	2018	
Fox S, 2013, HLTH ONLINE 2013, V0, P0	2013	20.07	**2015**	2018	
Kontos Emily, 2014, J Med Internet Res, V16, P0, DOI 10.2196/jmir.3117, DOI	2014	19.02	**2015**	2019	
Sorensen K, 2012, BMC PUBLIC HEALTH, V12, P0, DOI 10.1186/1471-2458-12-80, DOI	2012	15.94	**2015**	2017	
Mitsutake S, 2012, J MED INTERNET RES, V14, P406, DOI 10.2196/jmir.1927, DOI	2012	7.78	**2015**	2017	
Ghaddar SF, 2012, J SCHOOL HEALTH, V82, P28, DOI 10.1111/j.1746-1561.2011.00664.x, DOI	2012	6.66	**2015**	2017	
Yamin CK, 2011, ARCH INTERN MED, V171, P568, DOI 10.1001/archinternmed.2011.34, DOI	2011	5.48	**2015**	2016	
Tennant B, 2015, J MED INTERNET RES, V17, P0, DOI 10.2196/jmir.3992, DOI	2015	29.52	**2016**	2021	
Diviani N, 2015, J MED INTERNET RES, V17, P0, DOI 10.2196/jmir.4018, DOI	2015	17.95	**2016**	2021	
Levy H, 2015, J GEN INTERN MED, V30, P284, DOI 10.1007/s11606-014-3069-5, DOI	2015	7.44	**2016**	2021	
Fiordelli M, 2013, J MED INTERNET RES, V15, P0, DOI 10.2196/jmir.2430, DOI	2013	6.57	**2016**	2017	
van der Vaart R, 2013, J MED INTERNET RES, V15, P0, DOI 10.2196/jmir.2428, DOI	2013	4.3	**2016**	2018	
Mitsutake S, 2016, J MED INTERNET RES, V18, P0, DOI 10.2196/jmir.5413, DOI	2016	21.02	**2017**	2021	
Chung SY, 2015, CIN-COMPUT INFORM NU, V33, P150, DOI 10.1097/CIN.0000000000000146, DOI	2015	14.27	**2017**	2021	
Soellner R, 2014, J MEDIA PSYCHOL-GER, V26, P29, DOI 10.1027/1864-1105/a000104, DOI	2014	14.23	**2017**	2019	
Diviani N, 2016, PATIENT EDUC COUNS, V99, P1017, DOI 10.1016/j.pec.2016.01.007, DOI	2016	8.93	**2017**	2019	
Sorensen K, 2015, EUR J PUBLIC HEALTH, V25, P1053, DOI 10.1093/eurpub/ckv043, DOI	2015	8.24	**2017**	2021	
Watkins I, 2014, J MED INTERNET RES, V16, P0, DOI 10.2196/jmir.3318, DOI	2014	7.59	**2017**	2018	
Nguyen Jennifer, 2016, JMIR Public Health Surveill, V2, P0, DOI 10.2196/publichealth.4967, DOI	2016	7	**2017**	2018	
Paige SR, 2017, PATIENT EDUC COUNS, V100, P320, DOI 10.1016/j.pec.2016.09.008, DOI	2017	13.2	**2018**	2021	
van der Vaart Rosalie, 2017, J Med Internet Res, V19, P0, DOI 10.2196/jmir.6709, DOI	2017	12.28	**2018**	2021	
Diviani N, 2017, J MED INTERNET RES, V19, P0, DOI 10.2196/jmir.6749, DOI	2017	10.9	**2018**	2021	
Perez GP, 2015, REV ESP SALUD PUBLIC, V89, P329, DOI 10.4321/S1135-57272015000300010, DOI	2015	9.74	**2018**	2021	
Richtering Sarah S, 2017, JMIR Hum Factors, V4, P0, DOI 10.2196/humanfactors.6217, DOI	2017	8.16	**2018**	2021	
Karnoe A, 2015, KNOW MANAGE E LEARN, V7, P576, DOI DOI 10.34105/J.KMEL.2015.07.038], DOI	2015	7.68	**2018**	2021	
Norgaard O, 2015, KNOW MAN E LEARN, V7, P522	2015	6.94	**2018**	2019	
Kruse CS, 2015, J MED INTERNET RES, V17, P70	2015	6.65	**2018**	2021	
Kayser Lars, 2015, JMIR Hum Factors, V2, P0, DOI 10.2196/humanfactors.3696, DOI	2015	6.36	**2018**	2019	
Hsu WC, 2014, J MED INTERNET RES, V16, P203, DOI 10.2196/jmir.3542, DOI	2014	6.36	**2018**	2019	
Park H, 2015, NURS EDUC TODAY, V35, P408, DOI 10.1016/j.nedt.2014.10.022, DOI	2015	6.13	**2018**	2021	
Kim H, 2017, PATIENT EDUC COUNS, V100, P1073, DOI 10.1016/j.pec.2017.01.015, DOI	2017	14.92	**2019**	2021	
Sudbury-Riley L, 2017, J MED INTERNET RES, V19, P0, DOI 10.2196/jmir.5998, DOI	2017	12.01	**2019**	2021	
Chung S, 2018, J MED INTERNET RES, V20, P0, DOI 10.2196/jmir.8759, DOI	2018	9.71	**2019**	2021	
Tubaishat A, 2016, NURS EDUC TODAY, V42, P47, DOI 10.1016/j.nedt.2016.04.003, DOI	2016	9.13	**2019**	2021	
Xesfingi S, 2016, INTERACT J MED RES, V5, P81, DOI 10.2196/ijmr.4749, DOI	2016	6.13	**2019**	2021	
