# Supplementary material for: Digital Health Literacy: Bibliometric Analysis
Source: J Med Internet Res. 2022 Jul 6;24(7):e35816. doi: 10.2196/35816 (PMC9301558; doi:10.2196/35816)
Supplement: Multimedia Appendix 1 [file jmir_v24i7e35816_app1.pdf]

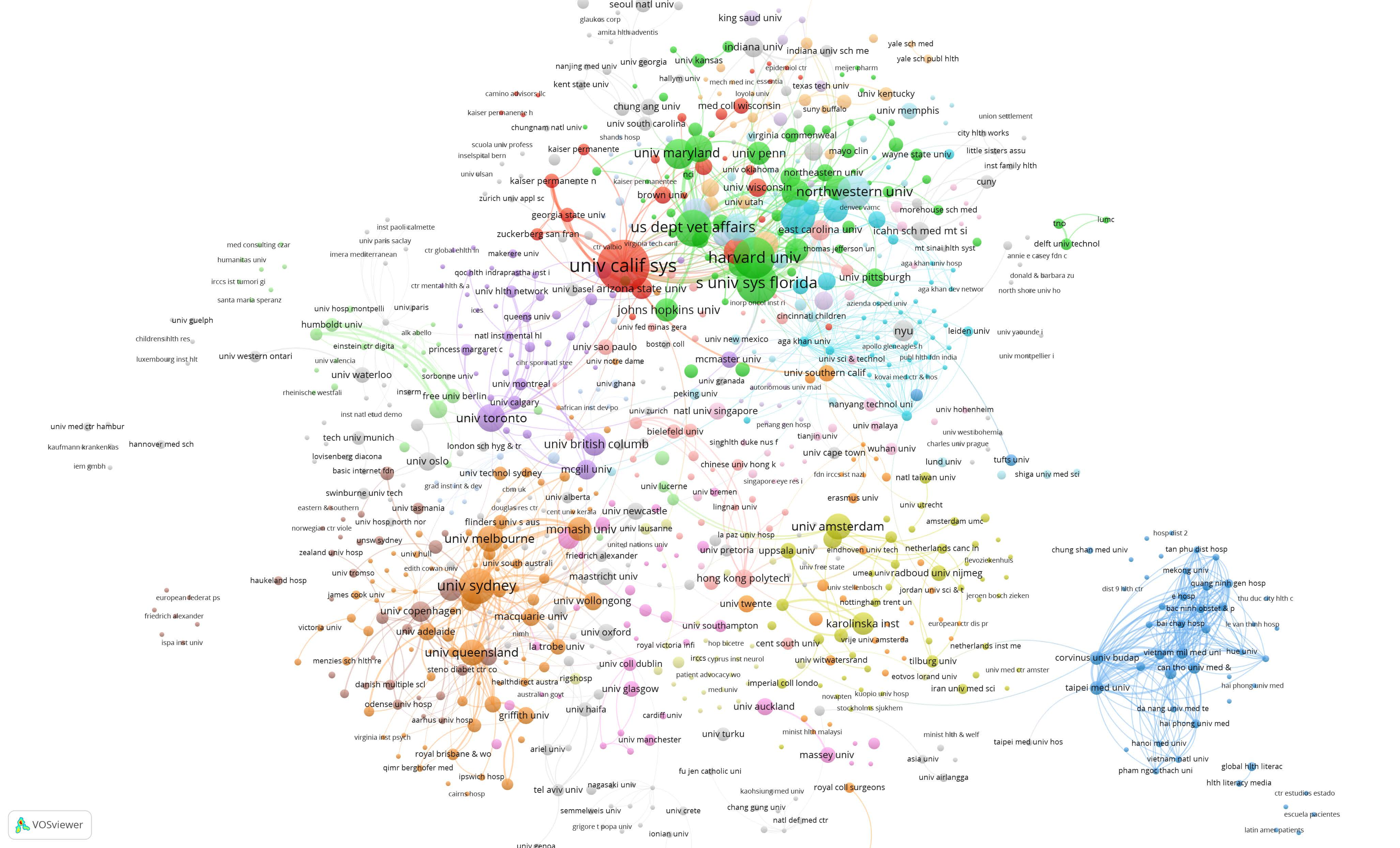

seoul natl univ
glaukos corp
king saud univ
amita hlth adventis
indiana univ
yale sch med
indiana univ sch me
yale sch publ hlth
nanjing med univ
univ georgia
univ kansas
epidemiol ctr
meijer pharm
kent state univ
hallym univ
mech med inc
essentia
texas tech univ
camino advisors llc
loyola univ
univ kentucky
chung ang univ
med coll wisconsin
suny buffalo
univ memphis
kaiser permanente h
union settlement
chungnam natl univ
univ south carolina
city hlth works
shands hosp
virginia commonweal
scuola univ profess
kaiser permanente
mayo clin
wayne state univ
little sisters assu
inselspital bern
univ maryland
univ penn
univ oklahoma
northeastern univ
inst family hlth
univ ulsan
nci
kaiser permanente n
kaiser permanentee
univ wisconsin
cuny
zurich univ appl sc
brown univ
northwestern univ
univ utah
denver vamc
morehouse sch med
georgia state univ
tno
lumc
inst paoli calmette
us dept vet affairs
east carolina univ
icahn sch med mt si
univ paris saclay
zuckerberg san fran
virginia tech caril
delft univ technol
med consulting czar
ctr valbio
ctr global ehlth in
thomas jefferson un
mt sinai hlth syst
humanitas univ
imera mediterranean
makerere univ
harvard univ
annie e casey fdn c
univ calif sys
aga khan univ hosp
qoc hlth indraprastha inst i
iraccs ist tumori gi
univ pittsburgh
donald & barbara zu
ctr mental hlth & a
s univ sys florida
santa maria speranz
univ basel
arizona state univ
aga khan dev networ
north shore univ ho
univ hlth network
inorp oncol inst ri
azienda osped univ
ices
univ hosp montpelli
univ paris
johns hopkins univ
queens univ
cincinnati children
univ guelph
humboldt univ
univ fed minas gera
nyu
leiden univ
univ yaounde i
alk abello
childrens hlth res
natl inst mental hl
einstein ctr digita
boston coll
univ new mexico
aga khan univ
apollo gleneagles h
princess margaret c
univ sao paulo
univ sci & technol
publ hlth fdn india
univ montpellier i
luxembourg inst hlt
univ western ontari
univ valencia
cihr spor natl stee
univ notre dame
mcmaster univ
univ waterloo
sorbonne univ
univ granada
univ southern calif
kovai med ctr & hos
univ montreal
univ ghana
autonomous univ mad
rheinische westfali
inserm
free univ berlin
peking univ
univ calgary
african inst dev po
univ zurich
natl univ singapore
nanyang technol uni
univ hohenheim
inst natl etud demo
univ toronto
univ med ctr hambur
penang gen hosp
univ malaya
bielefeld univ
univ west bohemia
tianjin univ
tech univ munich
singhlth duke nus f
charles univ prague
kaufmann krankenkas
hannover med sch
london sch hyg & tr
univ british columb
wuhan univ
univ cape town
lovisenberg diacona
lund univ
univ oslo
tufts univ
iem gmbh
chinese univ hong k
basic internet fdn
univ technol sydney
mcgill univ
fdn irccs ist nazl
shiga univ med sci
natl taiwan univ
singapore eye res i
grad inst int & dev
univ lucerne
cbm uk
univ bremen
swinburne univ tech
erasmus univ
univ alberta
univ tasmania
eastern & southern
lingnan univ
univ utrecht
douglas res ctr
univ newcastle
cent univ kerala
norwegian ctr viole
univ hosp north nor
flinders univ s aus
amsterdam umc
univ amsterdam
monash univ
univ lausanne
hosp dist 2
la paz univ hosp
unsw sydney
univ melbourne
united nations univ
tan phu dist hosp
zealand univ hosp
univ pretoria
uppsala univ
eindhoven univ tech
netherlands canc in
chung shan med univ
univ hull
friedrich alexander
flevoziekenhuis
univ south australi
mekong univ
edith cowan univ
univ free state
univ tromso
radboud univ nijmeg
umea univ
maastricht univ
hong kong polytech
haukeland hosp
quang ninh gen hosp
dist 9 hlth ctr
univ stellenbosch
jordan univ sci & t
univ sydney
european federat ps
e hosp
thu duc city hlth c
james cook univ
jeroen bosch zieken
univ wollongong
nottingham trent un
bac ninh obstet & p
univ twente
univ copenhagen
friedrich alexander
bai chay hosp
macquarie univ
le van thinh hosp
karolinska inst
european ctr dis pr
victoria univ
univ adelaide
univ southampton
nimh
univ oxford
vrije univ amsterda
ispa inst univ
netherlands inst me
royal victoria infi
hop bicetre
cent south univ
la trobe univ
vietnam mil med uni
hue univ
univ queensland
corvinus univ budap
irccs cyprus inst neurol
univ witwatersrand
tilburg univ
menzies sch hlth re
steno diabet ctr co
univ coll dublin
can tho univ med &
univ med ctr amster
patient advocacy wo
eotvos lorand univ
healthdirect austra
rigshosp
danish multiple scl
imperial coll londo
hai phong univ med
taipei med univ
iran univ med sci
med univ
novapten
univ glasgow
kuopio univ hosp
australian govt
odense univ hosp
da nang univ med te
stockholms sjukhem
univ auckland
univ haifa
cardiff univ
hai phong univ med
griffith univ
aarhus univ hosp
minist hlth malaysi
univ turku
minist hlth & welf
virginia inst psych
taipei med univ hos
hanoi med univ
univ manchester
massey univ
ariel univ
vietnam natl univ
global hlth literac
asia univ
royal brisbane & wo
pham ngoc thach uni
qimr berghofer med
fu jen catholic uni
univ airlangga
hlth literacy media
ipswich hosp
nagasaki univ
ctr estudios estado
tel aviv univ
royal coll surgeons
kaohsiung med univ
cairns hosp
escuela pacientes
semmelweis univ
univ crete
chang gung univ
natl def med ctr
latin amer patients
grigore t popa univ
ionian univ
univ genoa
VOSviewer
