# Supplementary material for: Digital Health Literacy: Bibliometric Analysis
Source: J Med Internet Res. 2022 Jul 6;24(7):e35816. doi: 10.2196/35816 (PMC9301558; doi:10.2196/35816)
Supplement: Multimedia Appendix 2 [file jmir_v24i7e35816_app2.pdf]

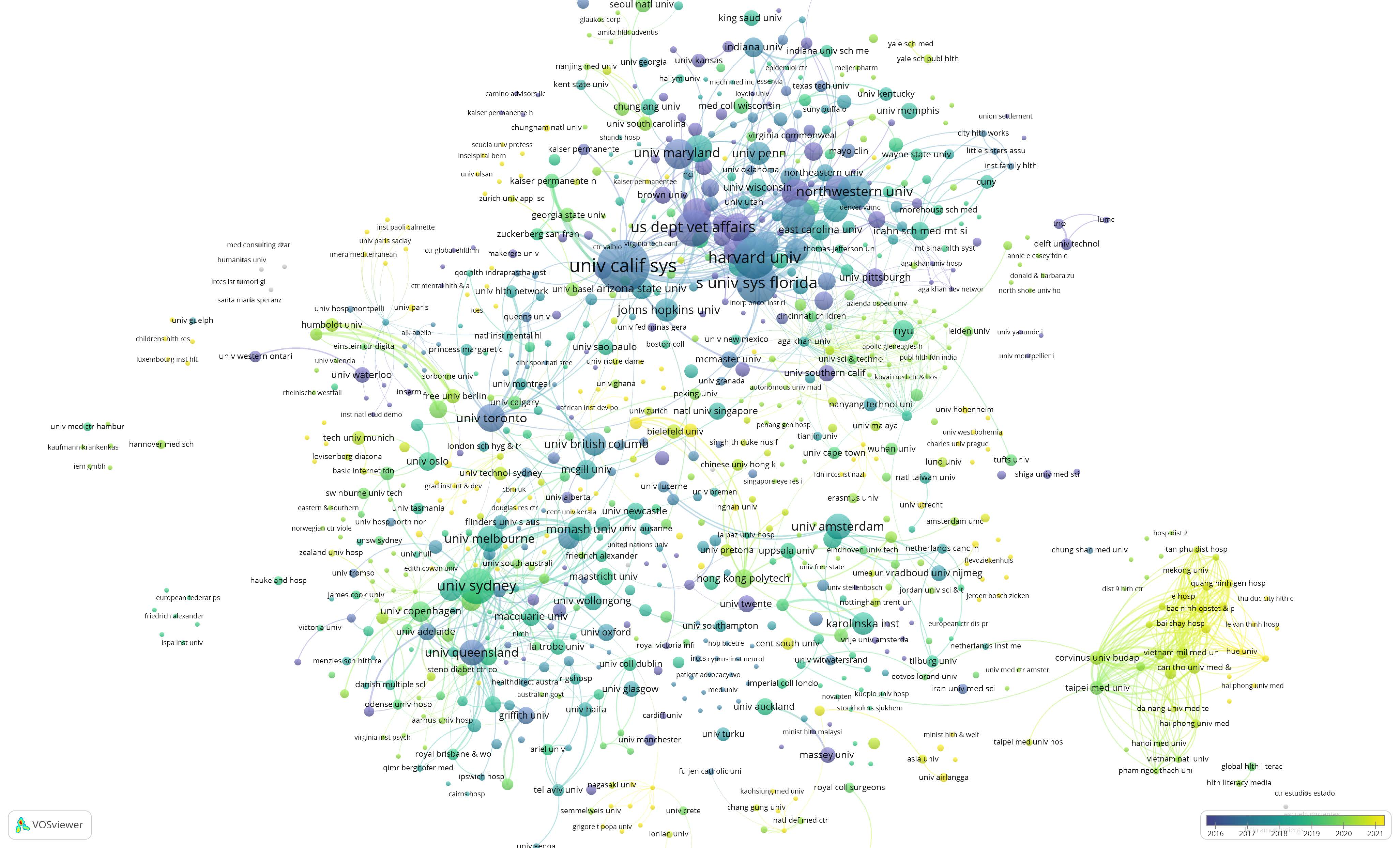
seoul natl univ
glaukos corp
amita hlth adventis
king saud univ
indiana univ
indiana univ sch me
yale sch med
yale sch publ hlth
nanjing med univ
univ georgia
univ kansas
epidemiol ctr
meijer pharm
hallym univ
mech med inc
essentia
texas tech univ
kent state univ
loyola univ
univ kentucky
camino advisors llc
chung ang univ
med coll wisconsin
suny buffalo
univ memphis
kaiser permanente h
union settlement
chungnam natl univ
univ south carolina
shands hosp
virginia commonweal
city hlth works
scuola univ profess
inselspital bern
kaiser permanente
univ maryland
univ penn
mayo clin
wayne state univ
little sisters assu
univ oklahoma
northeastern univ
inst family hlth
univ ulsan
nci
kaiser permanente n
kaiser permanentee
univ wisconsin
cuny
zurich univ appl sc
brown univ
northwestern univ
univ utah
denver vamc
morehouse sch med
georgia state univ
tno
lumc
us dept vet affairs
east carolina univ
icahn sch med mt si
inst paoli calmette
zuckerberg san fran
univ paris saclay
delft univ technol
med consulting czar
ctr valbio
virginia tech caril
ctr global hlth in
thomas jefferson un
mt sinai hlth syst
imera mediterranean
makerere univ
humanitas univ
harvard univ
annie e casey fdn c
univ calif sys
aga khan univ hosp
qoc hlth indraprastha inst i
irccs ist tumori gi
univ pittsburgh
donald & barbara zu
s univ sys florida
ctr mental hlth & a
santa maria speranz
univ hlth network
univ basel
arizona state univ
aga khan dev networ
north shore univ ho
inorp oncol inst ri
univ hosp montpelli
univ paris
azienda osped univ
johns hopkins univ
ices
queens univ
cincinnati children
univ guelph
humboldt univ
univ fed minas gera
nyu
alk abello
natl inst mental hl
leiden univ
univ yaounde i
childrens hlth res
einstein ctr digita
univ new mexico
aga khan univ
boston coll
univ sao paulo
apollo gleneagles h
princess margaret c
luxembourg inst hlt
univ western ontari
univ valencia
mcmaster univ
univ sci & technol
publ hlth fdn india
univ montpellier i
cihr sporinatl stee
univ notre dame
univ southern calif
univ waterloo
sorbonne univ
univ granada
kovai med ctr & hos
univ montreal
univ ghana
rheinische westfali
autonomous univ mad
inserm
free univ berlin
peking univ
univ calgary
nanyang technol uni
univ hohenheim
inst natl etud demo
african inst dev po
univ zurich
natl univ singapore
univ toronto
penang gen hosp
univ malaya
univ med ctr hambur
bielefeld univ
univ west bohemia
tianjin univ
tech univ munich
singhlth duke nus f
charles univ prague
kaufmann krankenkas
london sch hyg & tr
univ british columb
hannover med sch
univ cape town
wuhan univ
lovisenberg diacona
lund univ
tufts univ
univ oslo
iem gmbh
basic internet fdn
chinese univ hong k
univ technol sydney
mcgill univ
fdn irccs ist nazl
shiga univ med sci
singapore eye res i
natl taiwan univ
grad inst int & dev
univ lucerne
cbm uk
swinburne univ tech
univ bremen
univ alberta
erasmus univ
lingnan univ
eastern & southern
univ tasmania
univ utrecht
douglas res ctr
cent univ kerala
univ newcastle
univ hosp north nor
amsterdam umc
flinders univ s aus
norwegian ctr viole
univ lausanne
univ amsterdam
monash univ
hosp dist 2
la paz univ hosp
unsw sydney
univ melbourne
united nations univ
tan phu dist hosp
chung shan med univ
univ pretoria
uppsala univ
eindhoven univ tech
netherlands canc in
zealand univ hosp
friedrich alexander
univ hull
mekong univ
flevoziekenhuis
univ south australi
univ free state
edith cowan univ
umea univ
radboud univ nijmeg
univ tromso
maastricht univ
hong kong polytech
quang ninh gen hosp
haukeland hosp
dist 9 hlth ctr
univ stellenbosch
univ sydney
jordan univ sci & t
european federat ps
e hosp
thu duc city hlth c
jeroen bosch zieken
james cook univ
univ wollongong
univ twente
nottingham trent un
bac ninh obstet & p
univ copenhagen
friedrich alexander
macquarie univ
bai chay hosp
karolinska inst
le van thinh hosp
univ adelaide
european ctr dis pr
univ southampton
victoria univ
nimh
univ oxford
vrije univ amsterda
ispa inst univ
royal victoria infi
hop bicetre
netherlands inst me
univ queensland
cent south univ
la trobe univ
vietnam mil med uni
hue univ
corvinus univ budap
irccs cyprus inst neurol
menzies sch hlth re
univ witwatersrand
tilburg univ
steno diabet ctr co
univ coll dublin
can tho univ med &
univ med ctr amster
rigshosp
patient advocacy wo
eotvos lorand univ
healthdirect austra
imperial coll londo
hai phong univ med
danish multiple scl
taipei med univ
univ glasgow
iran univ med sci
australian govt
med univ
novapten
kuopio univ hosp
odense univ hosp
univ haifa
da nang univ med te
cardiff univ
univ auckland
stockholms sjukhem
aarhus univ hosp
griffith univ
hai phong univ med
univ turku
minist hlth malaysi
minist hlth & welf
virginia inst psych
taipei med univ hos
hanoi med univ
univ manchester
ariel univ
massey univ
asia univ
vietnam natl univ
royal brisbane & wo
global hlth literac
fu jen catholic uni
pham ngoc thach uni
qimr berghofer med
ipswich hosp
univ airlangga
hlth literacy media
nagasaki univ
kaohsiung med univ
royal coll surgeons
cairns hosp
tel aviv univ
ctr estudios estado
chang gung univ
semmelweis univ
univ crete
natl def med ctr
grigore t popa univ
ionian univ
univ genoa
VOSviewer
2016
2017
2018
2019
2020
2021
