# Supplementary material for: Digital Health Literacy: Bibliometric Analysis
Source: J Med Internet Res. 2022 Jul 6;24(7):e35816. doi: 10.2196/35816 (PMC9301558; doi:10.2196/35816)
Supplement: Multimedia Appendix 3 [file jmir_v24i7e35816_app3.pdf]

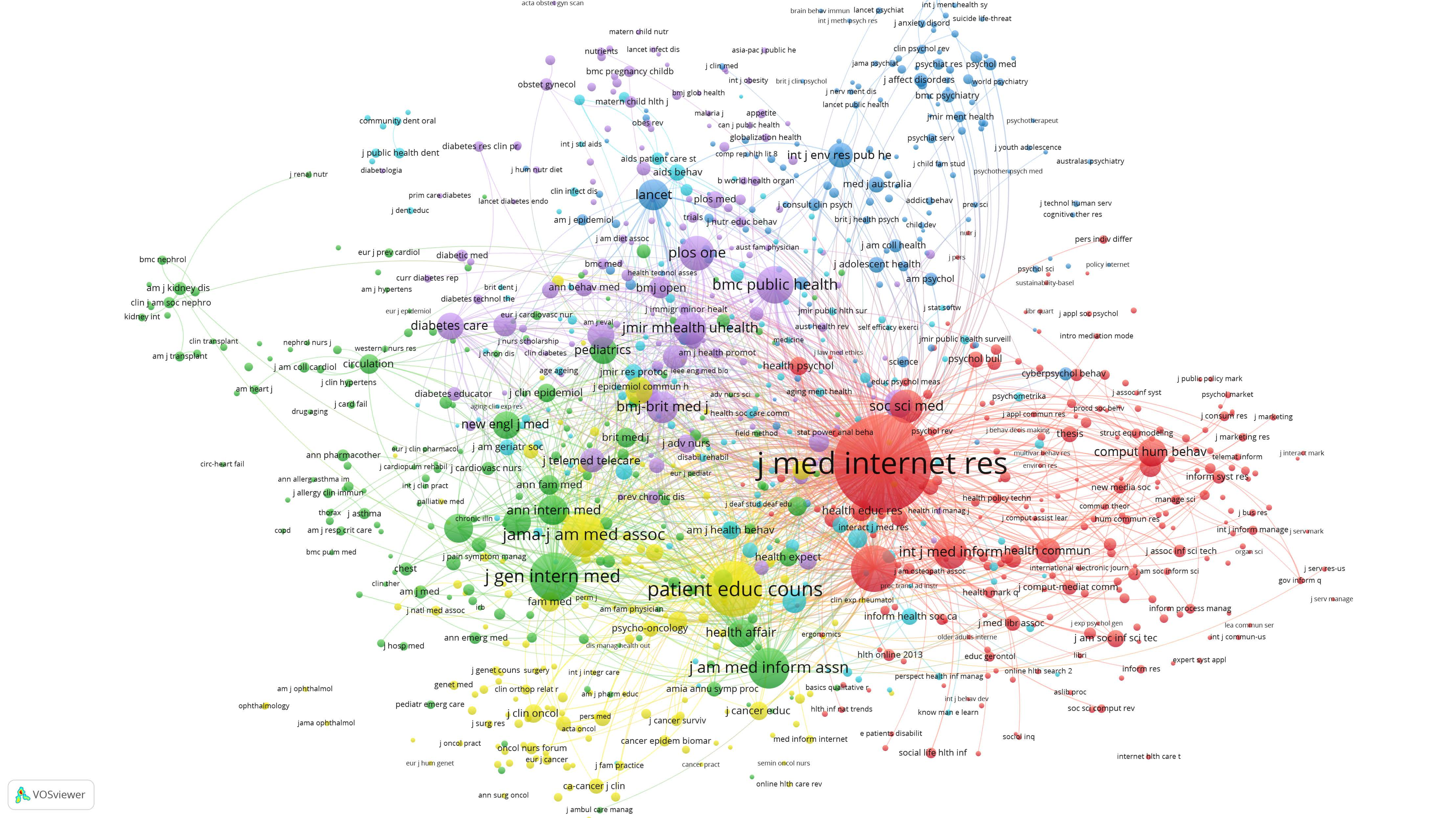
acta obstet gyn scan
brain behav immun
lancet psychiat
int j ment health sy
int j meth psych res
j anxiety disord
suicide life-threat
matern child nutr
nutrients
lancet infect dis
asia-pac j public he
clin psychol rev
jama psychiat
psychiat res
psychol med
bmc pregnancy childb
j clin med
obstet gynecol
int j obesity
brit j clin psychol
j affect disorders
world psychiatry
matern child hlth j
bmj glob health
j nerv ment dis
bmc psychiatry
lancet public health
malaria j
appetite
obes rev
can j public health
jmir ment health
psychotherapeut
community dent oral
globalization health
psychiat serv
int j std aids
j youth adolescence
diabetes res clin pr
comp rep hlth lit 8
int j env res pub he
j public health dent
aids patient care st
j child fam stud
australas psychiatry
diabetologia
j hum nutr diet
aids behav
psychother psych med
j renal nutr
b world health organ
med j australia
clin infect dis
lancet
prim care diabetes
plos med
addict behav
lancet diabetes endo
j consult clin psych
prev sci
j technol human serv
j dent educ
trials
am j epidemiol
j nutr educ behav
brit j health psych
cognitive ther res
child dev
nutr j
j am diet assoc
pers indiv differ
j am coll health
aust fam physician
eur j prev cardiol
plos one
j pers
bmc nephrol
diabetic med
j adolescent health
psychol sci
policy internet
bmc med
health technol asses
sustainability-basel
curr diabetes rep
am psychol
bmc public health
am j kidney dis
am j hypertens
brit dent j
ann behav med
bmj open
diabetes technol the
j stat softw
clin j am soc nephro
j immigr minor healt
jmir public hlth sur
libr quart
j appl soc psychol
eur j epidemiol
eur j cardiovasc nur
kidney int
diabetes care
am j eval
jmir mhealth uhealth
aust health rev
self efficacy exerci
clin transplant
j nurs scholarship
intro mediation mode
jmir public health surveill
nephrol nurs j
medicine
western j nurs res
am j transplant
j chron dis
clin diabetes
pediatrics
am j health promot
j law med ethics
science
psychol bull
j am coll cardiol
circulation
age ageing
jmir res protoc
ieee eng med bio
health psychol
cyberpsychol behav
j clin hypertens
j epidemiol commun h
educ psychol meas
j public policy mark
am heart j
diabetes educator
j clin epidemiol
adv nurs sci
aging ment health
j assoc inf syst
psychol market
j card fail
aging clin exp res
psychometrika
drug aging
bmj-brit med j
soc sci med
procd soc behv
j appl commun res
j consum res
j marketing
health soc care comm
new engl j med
j behav decis making
thesis
struct equ modeling
j marketing res
field method
stat power anal beha
psychol rev
brit med j
j adv nurs
eur j clin pharmacol
j am geriatr soc
multivar behav res
comput hum behav
ann pharmacother
telemat inform
j interact mark
circ-heart fail
j cardiopulm rehabil
disabil rehabil
j telemed telecare
j cardiovasc nurs
j med internet res
environ res
ann allerg asthma im
eur j pediatr
inform syst res
int j clin pract
ann fam med
new media soc
j allergy clin immun
palliative med
prev chronic dis
manage sci
health policy techn
j deaf stud deaf edu
commun theor
thorax
j asthma
ann intern med
health educ res
health inf manag j
j bus res
chronic illn
j comput assist lear
hum commun res
copd
am j resp crit care
interact j med res
int j inform manage
j serv mark
jama-j am med assoc
am j health behav
int j med inform
health commun
j assoc inf sci tech
organ sci
bmc pulm med
j pain symptom manag
health expect
chest
international electronic journ
j serv res-us
j am osteopath assoc
j am soc inform sci
clin ther
j gen intern med
gov inform q
patient educ couns
proc transl ad instr
am j med
j comput-mediat comm
health mark q
perm j
fam med
clin exp rheumatol
j serv manage
j natl med assoc
am fam physician
irb
inform health soc ca
inform process manag
psycho-oncology
j med libr assoc
j exp psychol gen
lea commun ser
health affair
ergonomics
ann emerg med
older adults interne
j am soc inf sci tec
int j commun-us
dis manag health out
hlth online 2013
educ gerontol
libri
expert syst appl
j hosp med
j am med inform assn
j genet couns
surgery
int j integr care
perspect health inf manag
online hlth search 2
inform res
genet med
am j ophthalmol
amia annu symp proc
basics qualitative r
asilb proc
clin orthop relat r
am j pharm educ
int j behav dev
pediatr emerg care
j cancer educ
hlth inf nat trends
ophthalmology
know man e learn
soc sci comput rev
j clin oncol
j surg res
pers med
j cancer surviv
jama ophthalmol
acta oncol
e patients disabilit
cancer epidem biomar
med inform internet
socioi inq
j oncol pract
oncol nurs forum
social life hlth inf
eur j cancer
internet hlth care t
semin oncol nurs
eur j hum genet
j fam practice
cancer pract
ca-cancer j clin
online hlth care rev
ann surg oncol
j ambul care manag
VOSviewer
