# Supplementary material for: Digital Health Literacy: Bibliometric Analysis
Source: J Med Internet Res. 2022 Jul 6;24(7):e35816. doi: 10.2196/35816 (PMC9301558; doi:10.2196/35816)
Supplement: Multimedia Appendix 7 [file jmir_v24i7e35816_app7.pdf]

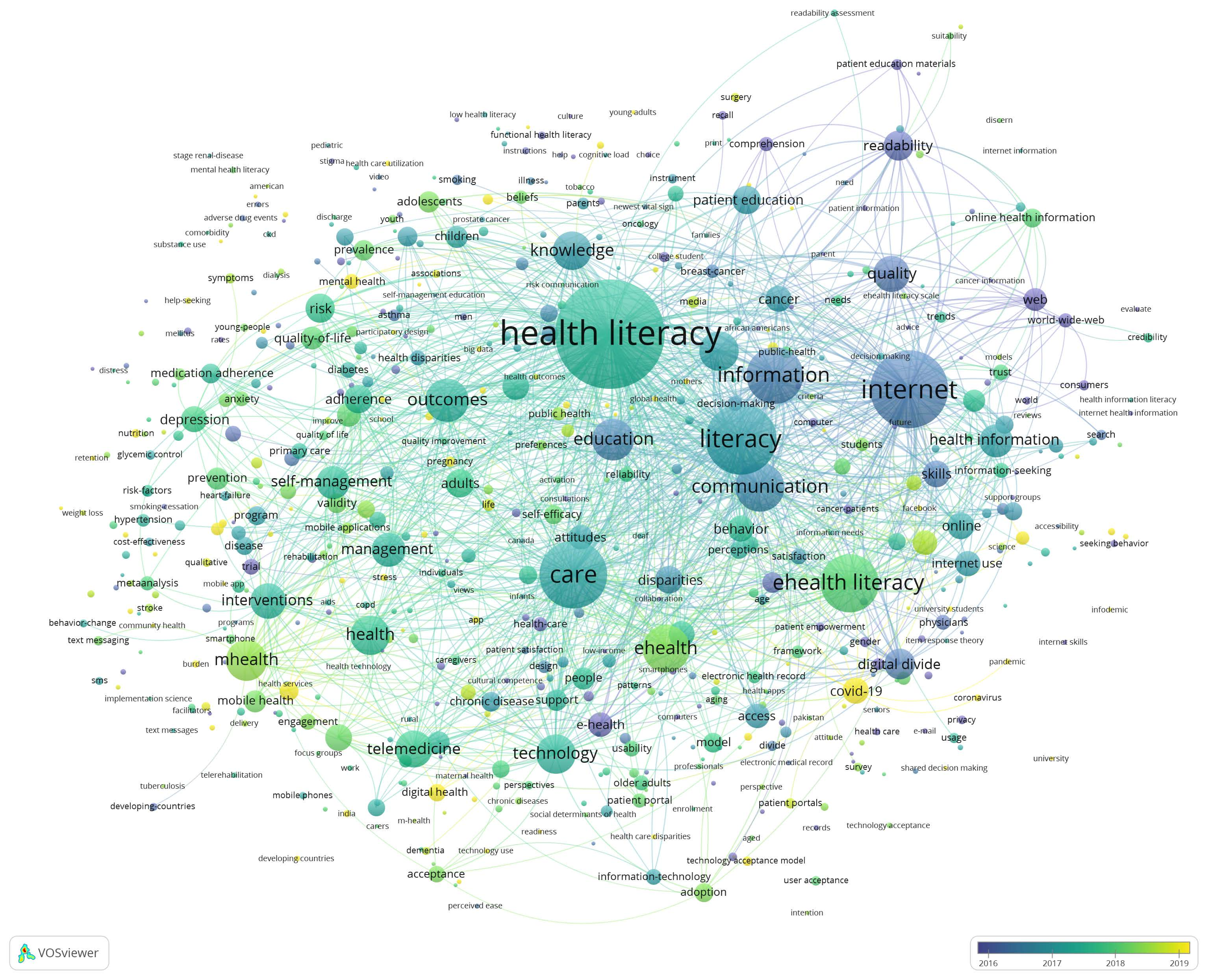
readability assessment
suitability
patient education materials
surgery
low health literacy
culture
young adults
recall
discern
functional health literacy
print
comprehension
readability
pediatric
instructions
help
cognitive load
choice
internet information
stage renal-disease
stigma
health care utilization
mental health literacy
video
smoking
illness
instrument
need
american
tobacco
beliefs
parents
newest vital sign
patient education
errors
adolescents
patient information
online health information
adverse drug events
discharge
youth
prostate cancer
oncology
comorbidity
ckd
children
families
substance use
prevalence
knowledge
college student
parent
breast-cancer
quality
symptoms
dialysis
mental health
associations
cancer information
risk communication
self-management education
media
cancer
needs
ehealth literacy scale
web
help-seeking
evaluate
risk
asthma
men
trends
young-people
african americans
advice
world-wide-web
mellitus
rates
quality-of-life
participatory design
health literacy
credibility
big data
public-health
health disparities
decision making
models
distress
medication adherence
diabetes
information
trust
health outcomes
mothers
internet
consumers
anxiety
adherence
outcomes
global health
criteria
world
health information literacy
decision-making
reviews
internet health information
improve
school
public health
computer
future
depression
nutrition
quality of life
education
literacy
search
quality improvement
preferences
students
health information
retention
glycemic control
primary care
pregnancy
reliability
skills
information-seeking
prevention
self-management
activation
risk-factors
heart-failure
adults
communication
smoking cessation
validity
life
consultations
support groups
weight loss
program
self-efficacy
cancer-patients
facebook
hypertension
mobile applications
behavior
accessibility
deaf
information needs
online
cost-effectiveness
canada
attitudes
disease
management
perceptions
science
seeking behavior
qualitative
rehabilitation
satisfaction
trial
internet use
individuals
care
stress
disparities
metaanalysis
mobile app
ehealth literacy
views
interventions
aids
collaboration
infants
age
copd
stroke
university students
infodemic
behavior-change
community health
programs
app
health-care
physicians
patient empowerment
text messaging
smartphone
health
item response theory
internet skills
gender
patient satisfaction
low-income
ehealth
framework
mhealth
burden
caregivers
pandemic
health technology
design
smartphones
digital divide
electronic health record
sms
health services
cultural competence
people
patterns
health apps
covid-19
implementation science
chronic disease
support
aging
mobile health
coronavirus
facilitators
seniors
delivery
rural
computers
pakistan
privacy
text messages
engagement
access
e-health
health care
e-mail
attitude
usage
focus groups
telemedicine
technology
usability
model
divide
university
work
maternal health
professionals
electronic medical record
survey
shared decision making
tuberculosis
telerehabilitation
perspectives
older adults
perspective
mobile phones
digital health
chronic diseases
patient portal
patient portals
developing-countries
india
enrollment
social determinants of health
carers
m-health
records
technology acceptance
readiness
health care disparities
aged
dementia
technology use
developing countries
technology acceptance model
acceptance
information-technology
user acceptance
adoption
perceived ease
intention
VOSviewer
2016
2017
2018
2019
