# Supplementary material for: Digital Health Literacy: Bibliometric Analysis
Source: J Med Internet Res. 2022 Jul 6;24(7):e35816. doi: 10.2196/35816 (PMC9301558; doi:10.2196/35816)
Supplement: Multimedia Appendix 9 [file jmir_v24i7e35816_app9.pdf]

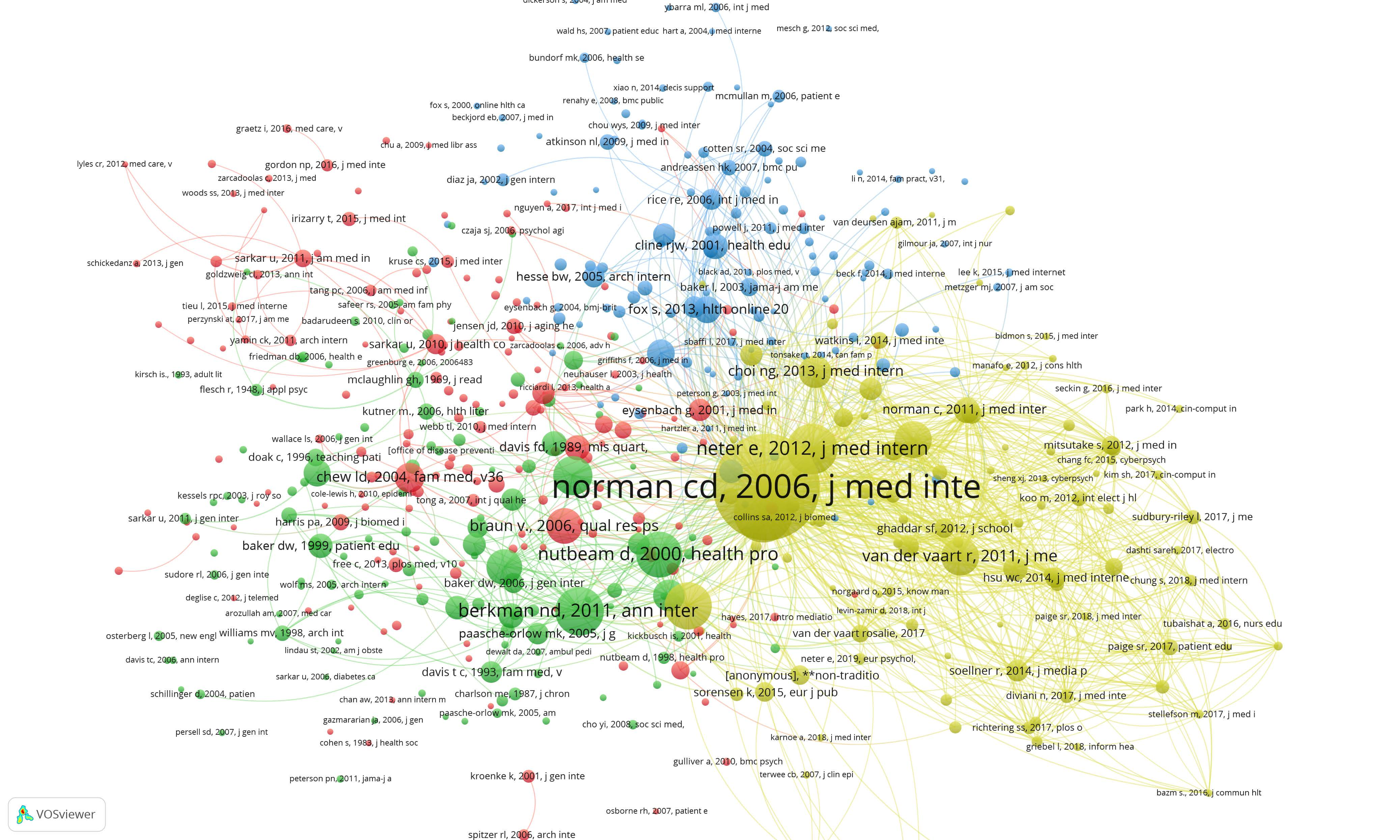
ybarra ml, 2006, int j med
wald hs, 2007, patient educ
hart a, 2004, j med interne
mesch g, 2012, soc sci med,
bundorf mk, 2006, health se
xiao n, 2014, decis support
mcmullan m, 2006, patient e
fox s, 2000, online hlth ca
renahy e, 2008, bmc public
beckjord eb, 2007, j med in
chou wys, 2009, j med inter
graetz i, 2016, med care, v
chu a, 2009, j med libr ass
atkinson nl, 2009, j med in
cotten sr, 2004, soc sci me
lyles cr, 2012, med care, v
gordon np, 2016, j med inte
andreassen hk, 2007, bmc pu
zarcadoolas c, 2013, j med
diaz ja, 2002, j gen intern
li n, 2014, fam pract, v31,
woods ss, 2013, j med inter
rice re, 2006, int j med in
nguyen a, 2017, int j med i
irizarry t, 2015, j med int
van deursen ajam, 2011, j m
czaja sj, 2006, psychol agi
powell j, 2011, j med inter
gilmour ja, 2007, int j nur
cline rjw, 2001, health edu
sarkar u, 2011, j am med in
kruse cs, 2015, j med inter
schickedanz a, 2013, j gen
black ad, 2011, plos med, v
beck f, 2014, j med interne
lee k, 2015, j med internet
hesse bw, 2005, arch intern
goldzweig cl, 2013, ann int
baker l, 2003, jama-j am me
metzger mj, 2007, j am soc
tang pc, 2006, j am med inf
tieu l, 2015, j med interne
safeer rs, 2005, am fam phy
eysenbach g, 2004, bmj-brit
fox s, 2013, hlth online 20
perzynski at, 2017, j am me
badarudeen s, 2010, clin or
jensen jd, 2010, j aging he
bidmon s, 2015, j med inter
yamin ck, 2011, arch intern
sarkar u, 2010, j health co
zarcadoolas c, 2006, adv h
sbaffi l, 2017, j med inter
watkins i, 2014, j med inte
friedman db, 2006, health e
tonsaker t, 2014, can fam p
greenburg e, 2006, 2006483
griffiths f, 2006, j med in
manafo e, 2012, j cons hlth
kirsch is., 1993, adult lit
neuhauser l, 2003, j health
choi ng, 2013, j med intern
mclaughlin gh, 1969, j read
flesch r, 1948, j appl psyc
ricciardi l, 2013, health a
seckin g, 2016, j med inter
peterson g, 2003, j med int
park h, 2014, cin-comput in
kutner m., 2006, hlth liter
eysenbach g, 2001, j med in
norman c, 2011, j med inter
webb tl, 2010, j med intern
hartzler a, 2011, j med int
wallace ls, 2006, j gen int
mitsutake s, 2012, j med in
[office of disease preventi
davis fd, 1989, mis quart,
neter e, 2012, j med intern
doak c, 1996, teaching pati
chang fc, 2015, cyberpsych
chew ld, 2004, fam med, v36
sheng xj, 2013, cyberpsych
kim sh, 2017, cin-comput in
norman cd, 2006, j med inte
kessels rpc, 2003, j roy so
cole-lewis h, 2010, epidemi
tong a, 2007, int j qual he
koo m, 2012, int elect j hl
collins sa, 2012, j biomed
sarkar u, 2011, j gen inter
sudbury-riley l, 2017, j me
harris pa, 2009, j biomed i
braun v., 2006, qual res ps
ghaddar sf, 2012, j school
baker dw, 1999, patient edu
dashti sareh, 2017, electro
nutbeam d, 2000, health pro
van der vaart r, 2011, j me
free c, 2013, plos med, v10
sudore rl, 2006, j gen inte
hsu wc, 2014, j med interne
chung s, 2018, j med intern
wolf ms, 2005, arch intern
baker dw, 2006, j gen inter
norgaard o, 2015, know man
deglise c, 2012, j telemed
levin-zamir d, 2018, int j
berkman nd, 2011, ann inter
arozullah am, 2007, med car
hayes, 2017, intro mediatio
paige sr, 2018, j med inter
tubaishat a, 2016, nurs edu
osterberg l, 2005, new engl
williams mv, 1998, arch int
paasche-orlow mk, 2005, j g
kickbusch is, 2001, health
van der vaart rosalie, 2017
lindau st, 2002, am j obste
paige sr, 2017, patient edu
dewalt da, 2007, ambul pedi
davis tc, 2006, ann intern
nutbeam d, 1998, health pro
neter e, 2019, eur psychol,
davis t c, 1993, fam med, v
soellner r, 2014, j media p
sarkar u, 2006, diabetes ca
[anonymous], **non-traditio
schillinger d, 2004, patien
charlson me, 1987, j chron
sorensen k, 2015, eur j pub
diviani n, 2017, j med inte
chan aw, 2013, ann intern m
paasche-orlow mk, 2005, am
stellefson m, 2017, j med i
gazmararian ja, 2006, j gen
richtering ss, 2017, plos o
cho yi, 2008, soc sci med,
persell sd, 2007, j gen int
karnoe a, 2018, j med inter
griebel l, 2018, inform hea
cohen s, 1983, j health soc
gulliver a, 2010, bmc psych
terwee cb, 2007, j clin epi
kroenke k, 2001, j gen inte
peterson pn, 2011, jama-j a
bazm s., 2016, j commun hlt
osborne rh, 2007, patient e
spitzer rl, 2006, arch inte
VOSviewer
